# Supplementary material for: Inhibition of glycolysis-driven immunosuppression with a nano-assembly enhances response to immune checkpoint blockade therapy in triple negative breast cancer
Source: Nat Commun. 2023 Nov 2;14:7021. doi: 10.1038/s41467-023-42883-2 (PMC10622423; doi:10.1038/s41467-023-42883-2)
Supplement: Supplementary file 1 — Supplementary Information [file 41467_2023_42883_MOESM1_ESM.pdf]

Inhibition of glycolysis-driven immunosuppression with a nano-assembly enhances response to immune checkpoint blockade therapy in triple negative breast cancer

Xijiao Ren[1], Zhuo Cheng[2], Jinming He[2], Xuemei Yao[2], Yingqi Liu[2], Kaiyong Cai[1], Menghuan Li[2*], Yan Hu[1*], Zhong Luo[2*]

[1]Key Laboratory of Biorheological Science and Technology, Ministry of Education, Chongqing University, Chongqing 400044, P. R. China.

[2]School of Life Science, Chongqing University, Chongqing 400044, P. R. China.

Email:

Yan Hu: huyan303@cqu.edu.cn

Zhong Luo: luozhong918@cqu.edu.cn

Menghuan Li: menghuanli@cqu.edu.cn

Supplementary Information

Supplementary Table 1. The DNA sequence of the aptamers.

Name of aptamer	Sequence (5'-3')
PD-L1	CCAACGGTCCATGTGTTCAGACGAGACGAACTTATGCGTATA CATTGTCACTCCCCGCCATTTTTTTTT
CTLA-4	TCCCTACGGCGCTAACGATGGTGAAAATGGGCCTAGGGTGG ACGGTGCCACCGTGCTACAACTTTTTTT

Supplementary Table 2. The siRNA sequence of the PD-L1 gene.

Name	Sense (5,-3,)
siNC	CGGUGAGCCAGGCGUGCAAUU
siPD-L1	GGCUACGUCCAGGAGCGCACC

Supplementary Table 3. The Source of antibodies.

Name	Reactivity	Cat	dilution	Source
PC7-anti-CD45 antibody	Mouse	E-AB-F1136G	1:50	elabscience
APC-anti-CD45 antibody	Mouse	E-AB-F1136E	1:50	elabscience
APC-anti-CD3 antibody	Mouse	E-AB-F1013E	1:50	elabscience
APC-anti-CD11c antibody	Mouse	E-AB-F0991E	1:50	elabscience
PE-anti-CD8a antibody	Mouse	E-AB-F1104D	1:50	elabscience
FITC-anti-CD4 antibody	Mouse	E-AB-F1353C	1:50	elabscience
APC-anti-F4/80 antibody	Mouse	E-AB-F0995E	1:50	elabscience
PE-anti-CD206 antibody	Mouse	E-AB-F1135D	1:50	elabscience
FITC-anti-CD86 antibody	Mouse	E-AB-F0994C	1:50	elabscience
FITC-anti-IFN-γ antibody	Mouse	E-AB-F1101C	1:50	elabscience
PE-anti-IL-10 antibody	Mouse	E-AB-F1197D	1:50	elabscience
FITC-anti-CD44 antibody	Mouse	E-AB-F1100C	1:50	elabscience
PE-anti-MHC-II antibody	Mouse	E-AB-F0990D	1:50	elabscience
PE-anti-CD62L antibody	Mouse	E-AB-F1011D	1:50	elabscience
PE-anti-GR1 antibody	Mouse	E-AB-F1120D	1:50	elabscience
FITC-anti-CD11b antibody	Mouse	E-AB-F1081C	1:50	elabscience
APC-anti-Foxp3 antibody	Mouse	APC-65089	1:50	proteintech
PE-anti-CTLA-4 antibody	Mouse	ab210383	1:100	Abcam
APC-anti-CD25 antibody	Mouse	E-AB-F1102E	1:50	elabscience
PE-anti-CD25 antibody	Mouse	E-AB-F1102D	1:50	elabscience
APC-anti-CD62L antibody	Mouse	E-AB-F1011E	1:50	elabscience
APC-anti-CD197 antibody	Mouse	120107	1:50	Biolegend
anti-PD-L1 antibody	Mouse	ab213480	1:1000	Abcam
APC-anti-PD-L1 antibody	Mouse	ab272329	1:100	Abcam
FITC-anti-CTLA-4 antibody	Mouse	ab24935	1:100	Abcam
APC-anti-CTLA-4 antibody	Mouse	106309	1:50	Biolegend
anti-Bcl-2 antibody	Mouse	381702	1:1000	Zenbio
anti-Bax antibody	Mouse	R23596	1:1000	Zenbio
anti-β-Tubulin antibody	Mouse	M30109S	1:1000	Abmart
FITC-anti-CD206 antibody	Human	E-AB-F1161C	1:50	elabscience
PE-anti-CD14 antibody	Human	E-AB-F1209D	1:50	elabscience
FITC-anti-CD80 antibody	Human	375405	1:50	Biolegend
PE-anti-HLA-DR antibody	Human	E-AB-F1111D	1:50	elabscience
FITC-anti-CD11c antibody	Human	E-AB-F1118C	1:50	elabscience
APC-anti-CD45 antibody	Human	E-AB-F1137E	1:50	elabscience
FITC-anti-CD11b antibody	Human	E-AB-F1146C	1:50	elabscience
APC-anti-CD4 antibody	Human	E-AB-F1109E	1:50	elabscience
PE-anti-Foxp3 antibody	Human	364703	1:50	Biolegend
FITC-anti-CD25 antibody	Human	E-AB-F1194C	1:50	elabscience
PE-anti-CD62L antibody	Human	E-AB-F1051D	1:50	elabscience
APC-anti-CD45RA antibody	Human	E-AB-F1052E	1:50	elabscience
FITC-anti-CD197 antibody	Human	E-AB-F1159C	1:50	elabscience
FITC-anti-CD4 antibody	Human	E-AB-F1352C	1:50	elabscience
PE-anti-CD8a antibody	Human	E-AB-F1110D	1:50	elabscience
APC-anti-CD3 antibody	Human	E-AB-F1001E	1:50	elabscience
InVivoMAb anti-human PD-L1 (B7-H1)	Human	BE0285	50 $\mu g \cdot mL^{-1} \cdot g^{-1}$	bioxcell
InVivoMAb anti-human CTLA-4 (CD152)	Human	BE0190	50 $\mu g \cdot mL^{-1} \cdot g^{-1}$	bioxcell

Supplementary Table 4. The validation information of antibodies.

Name	Validation information
PC7-anti-CD45 antibody	https://www.elabscience.cn/p-pe_cyanine5_anti_mouse_cd45_antibody_30_f11_-172697.html
APC-anti-CD45 antibody	https://www.elabscience.cn/p-apc_anti_mouse_cd45_antibody_30_f11_-172695.html
APC-anti-CD3 antibody	https://www.elabscience.cn/p-apc_anti_mouse_cd3_antibody_17a2_-133030.html
APC-anti-CD11c antibody	https://www.elabscience.cn/p-apc_anti_mouse_cd11c_antibody_n418_-132974.html
PE-anti-CD8a antibody	https://www.elabscience.cn/p-pe_anti_mouse_cd8a_antibody_53_6.7_-133053.html
FITC-anti-CD4 antibody	https://www.elabscience.cn/p-fitc_anti_mouse_cd4_antibody_rm4_5_-531529.html
APC-anti-F4/80 antibody	https://www.elabscience.cn/p-apc_anti_mouse_f4_80_antibody_ci_a3_1_-132982.html
PE-anti-CD206 antibody	https://www.elabscience.cn/p-pe_anti_mouse_cd206_antibody_c068c2_-147870.html
FITC-anti-CD86 antibody	https://www.elabscience.cn/p-fitc_anti_mouse_cd86_antibody_gl_1_-149393.html
FITC-anti-IFN-γ antibody	https://www.elabscience.cn/p-fitc_anti_mouse_ifn_gamma_antibody_xmg1.2_-172590.html
PE-anti-IL-10 antibody	https://www.elabscience.cn/p-pe_anti_mouse_il_10_antibody_jes5_16e3_-172898.html
FITC-anti-CD44 antibody	https://www.elabscience.cn/p-fitc_anti_human_mouse_cd44_antibody_im7_-176555.html
PE-anti-MHC-II antibody	https://www.elabscience.cn/p-pe_anti_mouse_mhc_ii_i_a_i_e_antibody_m5_114_-132965.html
PE-anti-CD62L antibody	https://www.elabscience.cn/p-pe_anti_mouse_cd62l_antibody_mel14_-134631.html
PE-anti-GR1 antibody	https://www.elabscience.cn/p-pe_anti_mouse_ly_6g_ly_6c_gr_1_antibody_rb6_8c5_-150590.html
FITC-anti-CD11b antibody	https://www.elabscience.cn/p-fitc_anti_mouse_human_cd11b_antibody_m1_70_-133068.html
APC-anti-Foxp3 antibody	https://www.ptgcn.com/products/Foxp3-Antibody-APC-65089.htm
PE-anti-CTLA-4 antibody	https://www.abcam.cn/products/primary-antibodies/pe-ctla4-antibody-uc10-4f10-11-ab210383.html
APC-anti-CD25 antibody	https://www.elabscience.cn/p-apc_anti_mouse_cd25_antibody_pc_61.5.3_-134626.html
PE-anti-CD25 antibody	https://www.elabscience.cn/p-pe_anti_mouse_cd25_antibody_pc_61.5.3_-172594.html
APC-anti-CD62L antibody	https://www.elabscience.cn/p-apc_anti_mouse_cd62l_antibody_mel14_-172281.html
APC-anti-CD197 antibody	https://www.biolegend.com/en-us/products/apc-anti-mouse-cd197-ccr7-antibody-2822
anti-PD-L1 antibody	https://www.abcam.cn/products/primary-antibodies/pd-l1-antibody-epr20529-ab213480.html
APC-anti-PD-L1 antibody	https://www.abcam.cn/products/primary-antibodies/apc-pd-l1-antibody-10f9g2-ab272329.html
FITC-anti-CTLA-4 antibody	https://www.abcam.cn/products/primary-antibodies/fitc-ctla4-antibody-1b8-ab24935.html
APC-anti-CTLA-4 antibody	https://www.biolegend.com/en-us/products/apc-anti-mouse-cd152-antibody-5455
anti-Bcl-2 antibody	http://www.zen-bio.cn/prod_view.aspx?IsActiveTarget=True&TypeId=180&Id=539422&FId=t3:180:3
anti-Bax antibody	http://www.zen-bio.cn/prod_view.aspx?IsActiveTarget=True&TypeId=171&Id=533394&FId=t3:171:3
anti-β-Tubulin antibody	http://www.ab-mart.com.cn/page.aspx?node=%2059%20&id=%20992
FITC-anti-CD206 antibody	https://www.elabscience.cn/p-fitc_anti_human_cd206_antibody_15_2_-172796.html
PE-anti-CD14 antibody	https://www.elabscience.cn/p-pe_anti_human_cd14_antibody_m5e2_-172927.html
FITC-anti-CD80 antibody	https://www.biolegend.com/en-us/products/fitc-anti-human-cd80-antibody-20587
PE-anti-HLA-DR antibody	https://www.elabscience.cn/p-pe_anti_human_hla_dr_antibody_l243_-133109.html
FITC-anti-CD11c antibody	https://www.elabscience.cn/p-fitc_anti_human_cd11c_antibody_bu15_-133020.html
APC-anti-CD45 antibody	https://www.elabscience.cn/p-apc_anti_human_cd45_antibody_hi30_-172704.html
FITC-anti-CD11b antibody	https://www.elabscience.cn/p-fitc_anti_human_cd11b_antibody_icrf44_-172748.html
APC-anti-CD4 antibody	https://www.elabscience.cn/p-apc_anti_human_cd4_antibody_rpa_t4_-133094.html
PE-anti-Foxp3 antibody	https://www.biolegend.com/en-us/products/pe-anti-human-foxp3-recombinant-antibody-20719
FITC-anti-CD25 antibody	https://www.elabscience.cn/p-fitc_anti_human_cd25_antibody_bc96_-172888.html
PE-anti-CD62L antibody	https://www.elabscience.cn/p-pe_anti_human_cd62l_antibody_dreg56_-172422.html
APC-anti-CD45RA antibody	https://www.elabscience.cn/p-apc_anti_human_cd45ra_antibody_hi100_-134570.html
FITC-anti-CD197 antibody	https://www.elabscience.cn/p-fitc_anti_human_cd197_ccr7_antibody_g043h7_-172791.html
FITC-anti-CD4 antibody	https://www.elabscience.cn/p-fitc_anti_human_cd4_antibody_sk3_-531520.html
PE-anti-CD8a antibody	https://www.elabscience.cn/p-pe_anti_human_cd8a_antibody_okt_8_-133101.html
APC-anti-CD3 antibody	https://www.elabscience.cn/p-apc_anti_human_cd3_antibody_okt_3_-132990.html
InVivoMAb anti-human PD-L1 (B7-H1)	https://bioxcell.com/invivomab-anti-human-pd-l1-b7-h1
InVivoMAb anti-human CTLA-4 (CD152)	https://bioxcell.com/invivomab-anti-human-ctla-4-cd152-be0190

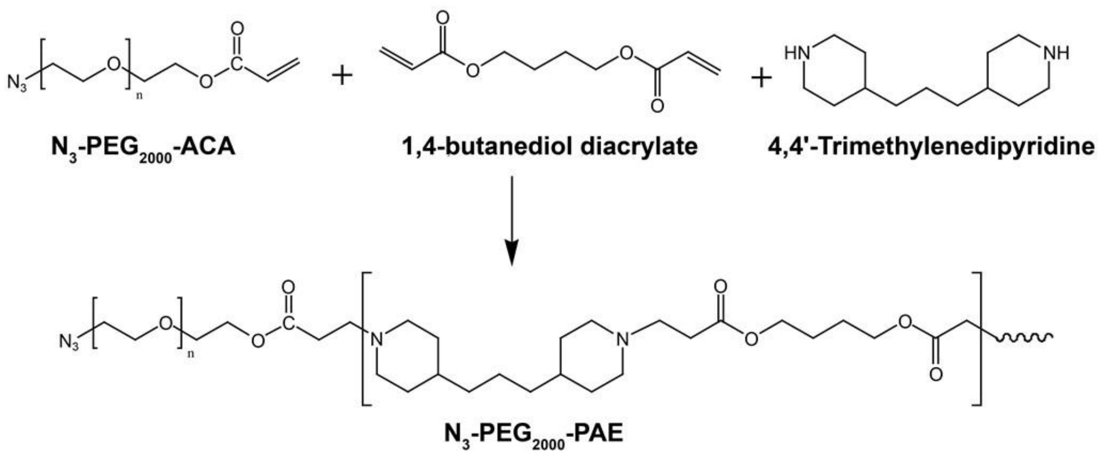

Supplementary Figure 1. Synthesis route of N_3-PEG2000-PAE (N_3-PEG2000-β amino ester).

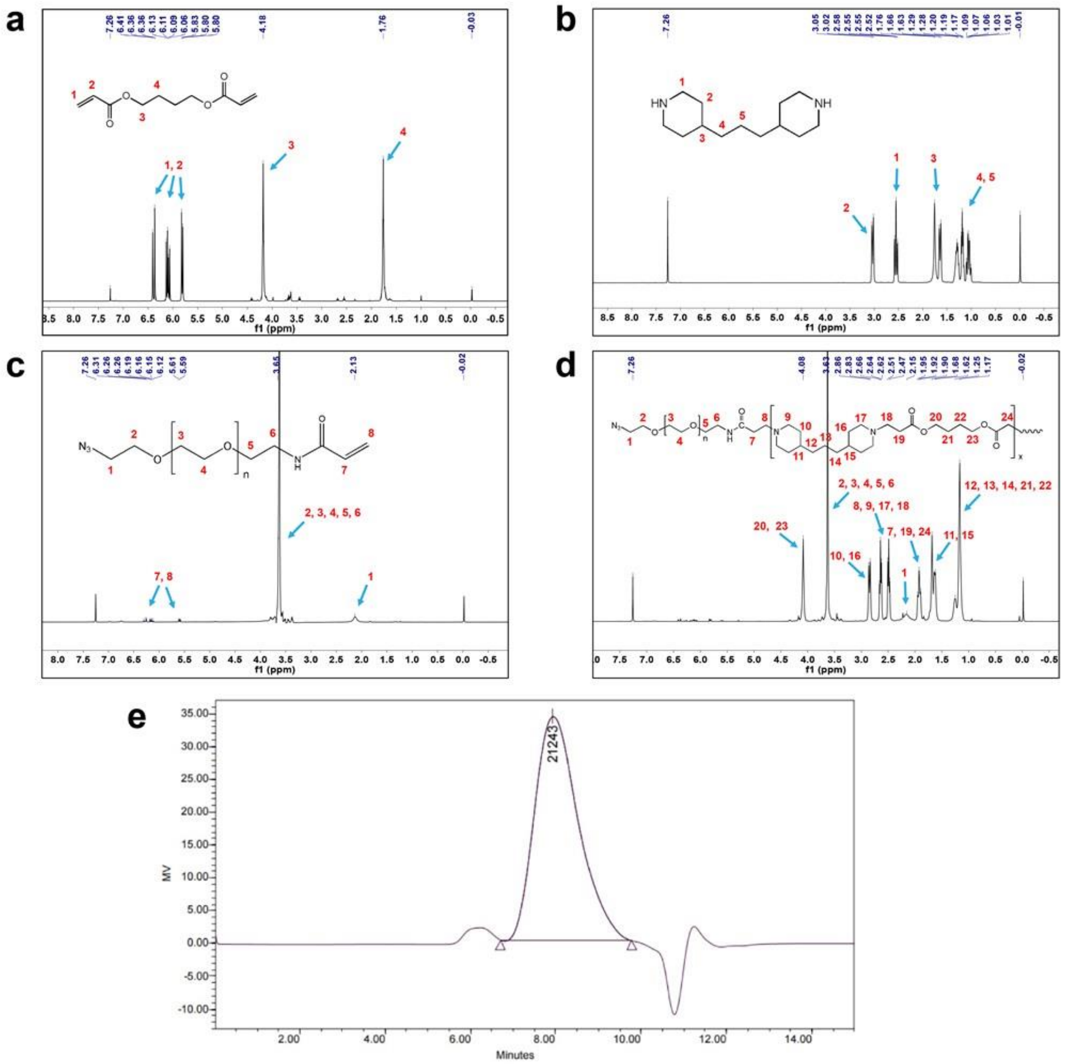

Supplementary Figure 2. H^1-NMR spectra of key products in DNA-PAE synthesis (400 MHz, Chloroform-*d*, ppm). (a) 1,4-butanediol diacrylate. δ 6.43-6.34 (m, 2H), 6.10 (dd, J = 17.3, 10.4 Hz, 2H), 5.87-5.75 (m, 2H), 4.18 (s, 4H), 1.76 (s, 4H). (b) 4,4'-trimethylenedipyridine. δ 3.03 (d, J = 12.1 Hz, 8H), 2.64-2.45 (m, 8H), 1.89-1.53 (m, 2H), 1.36-0.96 (m, 6H). (c) N_3-PEG_{2000}-ACA (N_3-PEG_{2000}-acrylamide). (d) N_3-PEG_{2000}-PAE (N_3-PEG_{2000}-β amino ester). (e) The molecular mass of N_3-PEG_{2000}-PAE by GPC.

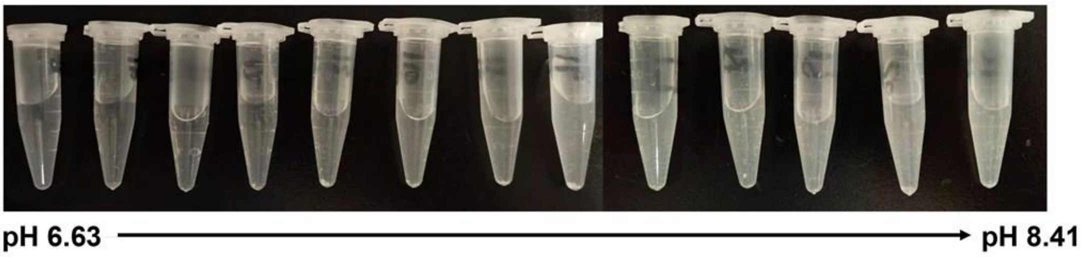

Supplementary Figure 3. N_3-PEG_{2000}-PAE solubility under different pH values.

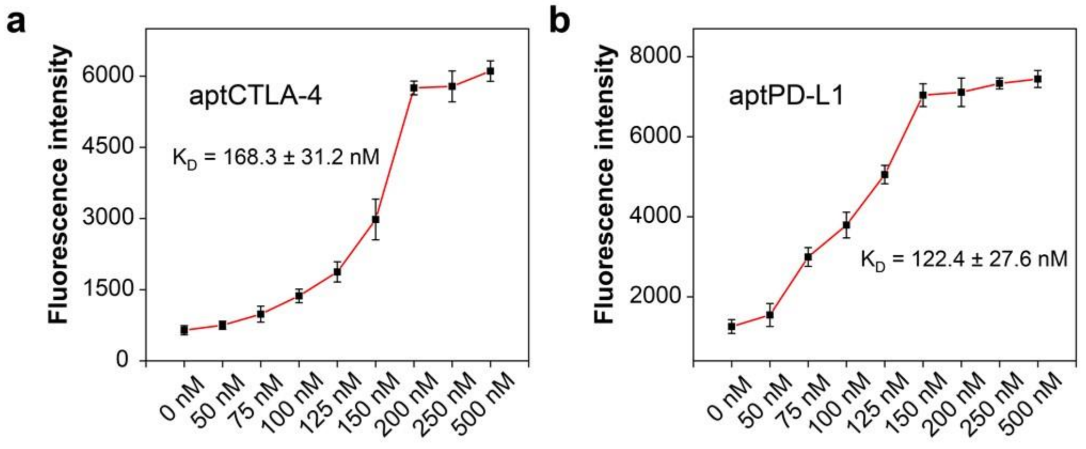

Supplementary Figure 4. Evaluation on the binding affinity of aptCTLA-4 and aptPD-L1 with their cognate receptors. (a) The dissociation constant of FAM-aptCTLA-4 to CTLA-4 under graded concentrations. (b) The dissociation constant of Cy5-aptPD-L1 to PD-L1 protein under graded concentrations. Data are presented as mean values ± SEM (n = 3 independent experiments for panels a-b). Source data are provided as a Source Data file.

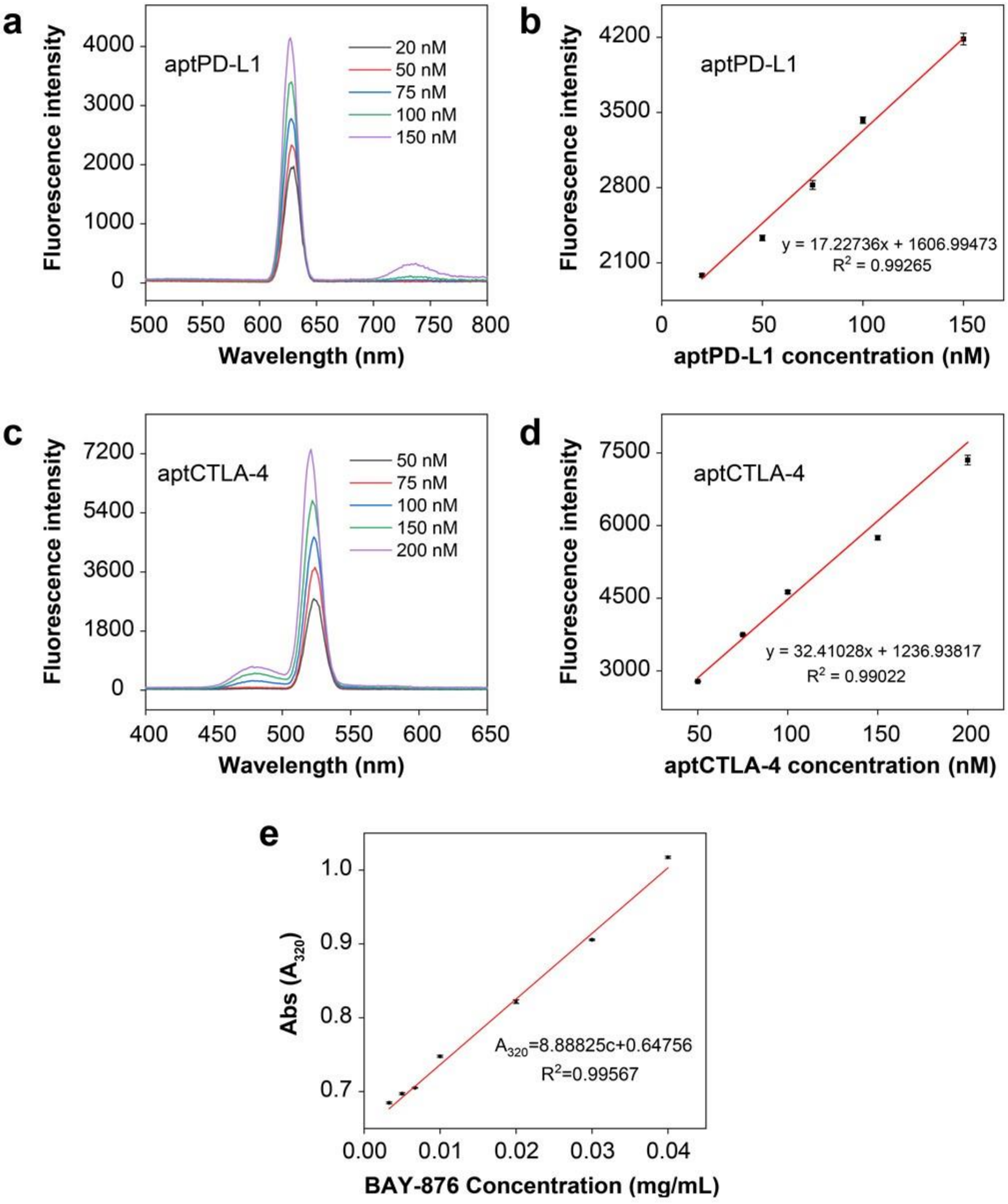

Supplementary Figure 5. Standard curves of the fluorescence intensity for Cy5-aptPD-L1 (a-b) and FAM-aptCTLA-4 (c-d) at different concentrations. (e) Standard curves of the UV-vis absorption for BAY-876 at different concentrations. The related experiments in panels a and c were repeated three times independently with similar results. Data are presented as mean values ± SEM (n = 3 independent experiments for panels b, d and e). Source data are provided as a Source Data file.

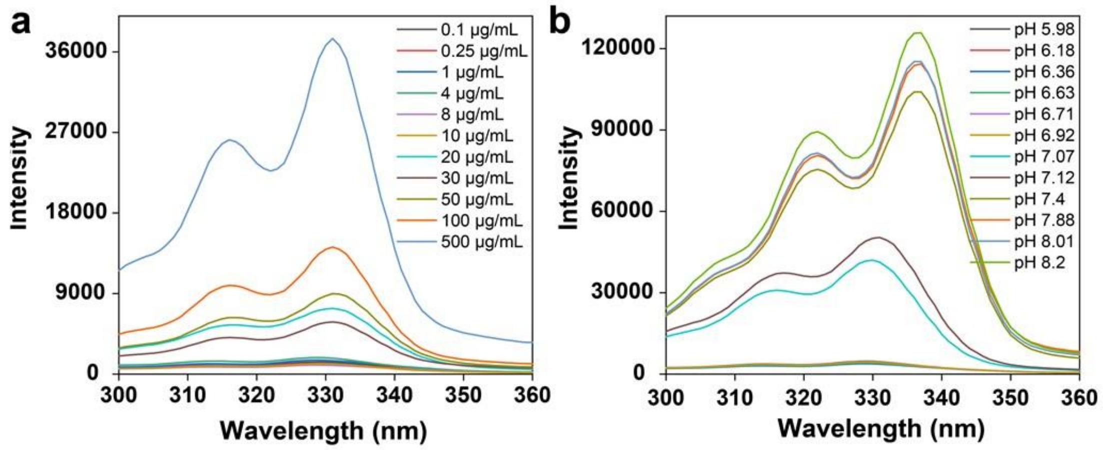

Supplementary Figure 6. Critical micellar concentration (a) and critical pH (b) of DNA-PAE nanoassemblies. The related experiments were repeated three times independently with similar results. Source data are provided as a Source Data file.

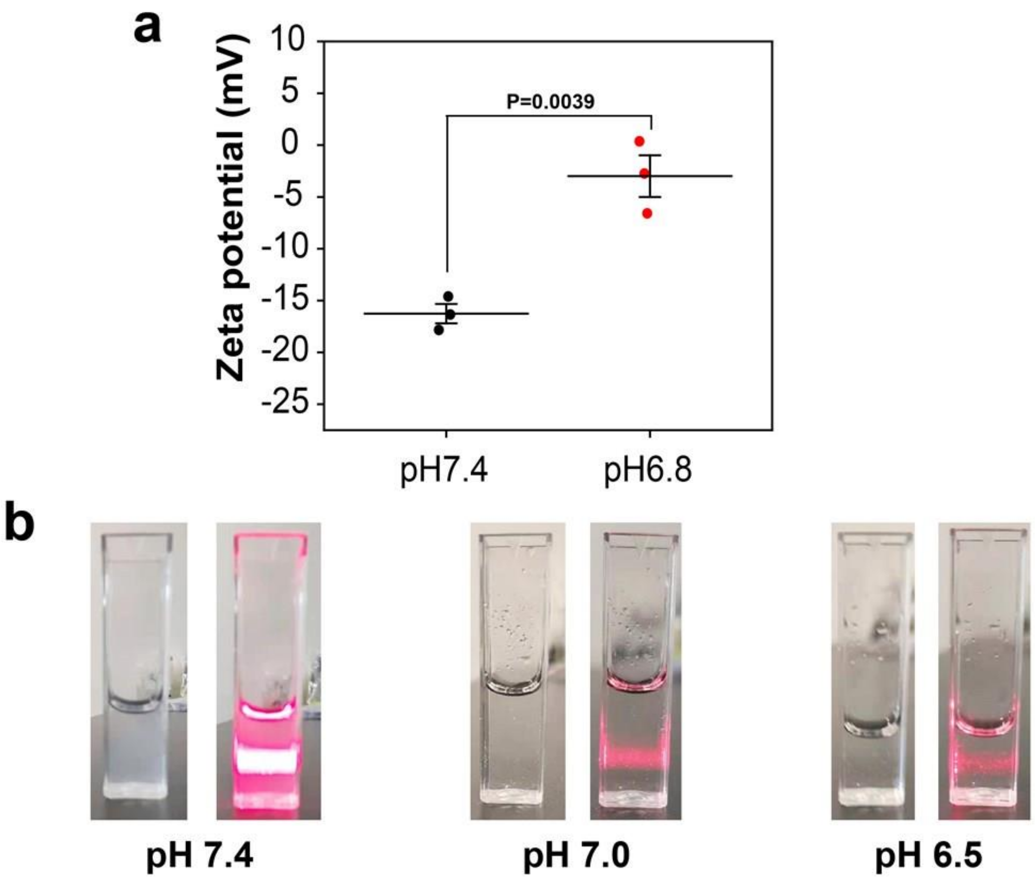

Supplementary Figure 7. Protonation of DNA-PAE nanoassemblies under acidic condition. (a) Zeta potential changes of DNA-PAE nanoassemblies at different pH conditions (n=3). (b) Tyndall effect of DNA-PAE nanoassemblies in PBS buffer at different pH values. Data are presented as mean values ± SEM (n = 3 independent experiments for panel a). Statistical analysis was carried out via one-way ANOVA method. Source data are provided as a Source Data file.

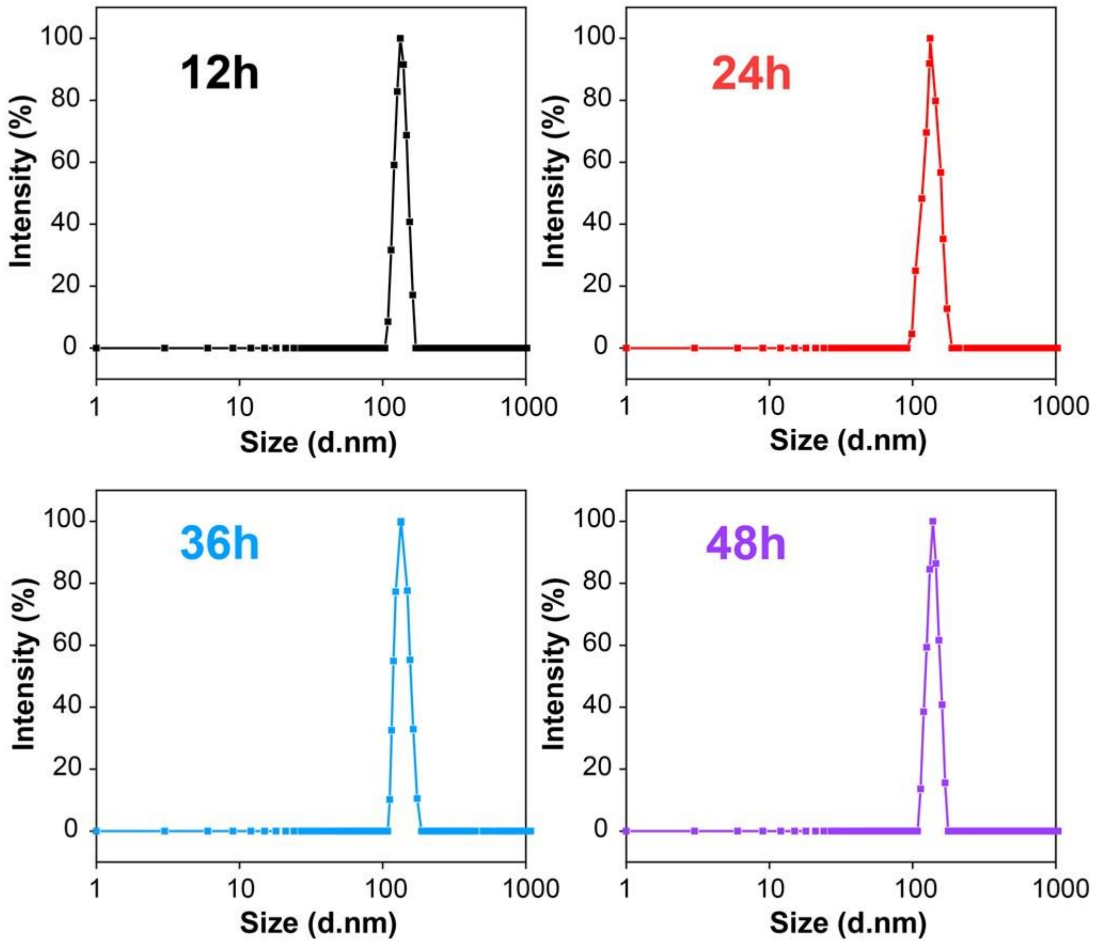

Supplementary Figure 8. Stability of DNA-PAE@BAY-876 nanoassemblies in pH 7.4 PBS solution. The related experiments were repeated three times independently with similar results. Source data are provided as a Source Data file.

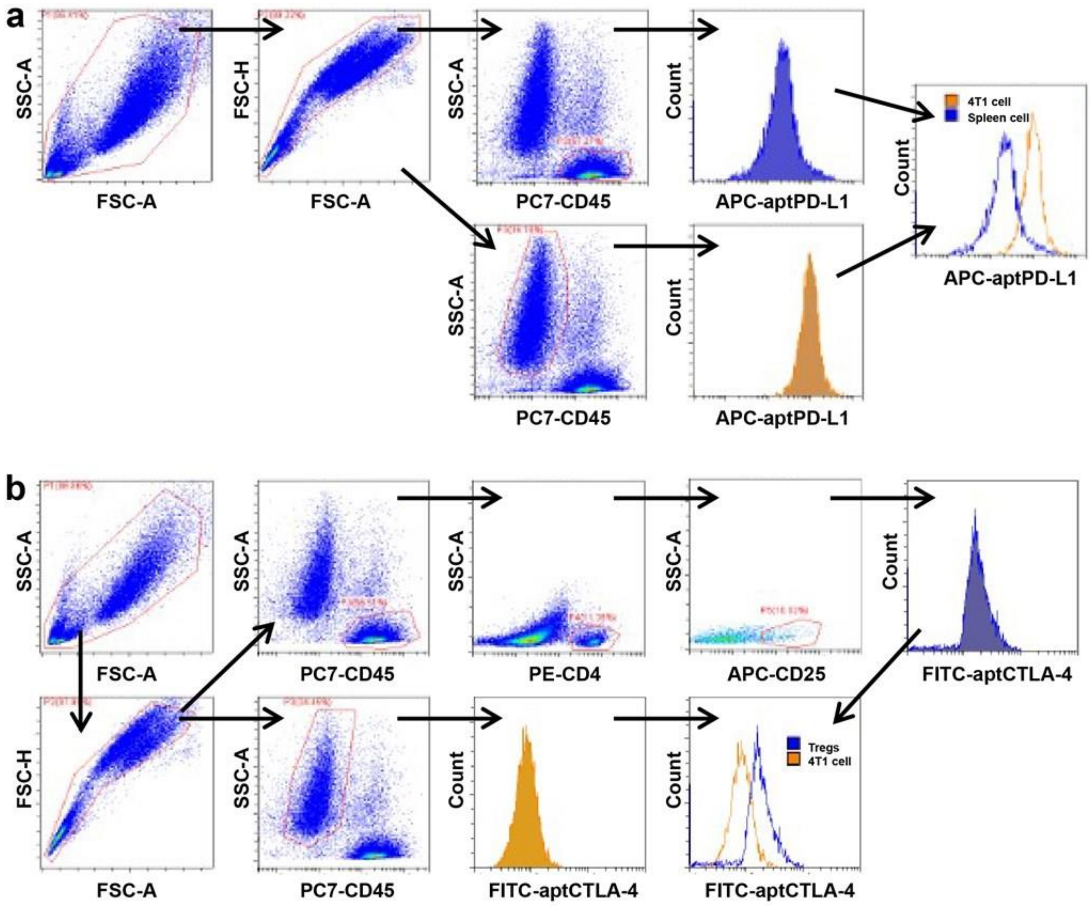

Supplementary Figure 9. Targeting ability of aptPD-L1 (a) and aptCTLA-4 (b) in the co-incubation system of 4T1 cells and splenic immune cells. Flow cytometry experiments in panels a and b were repeated three times independently with similar results.

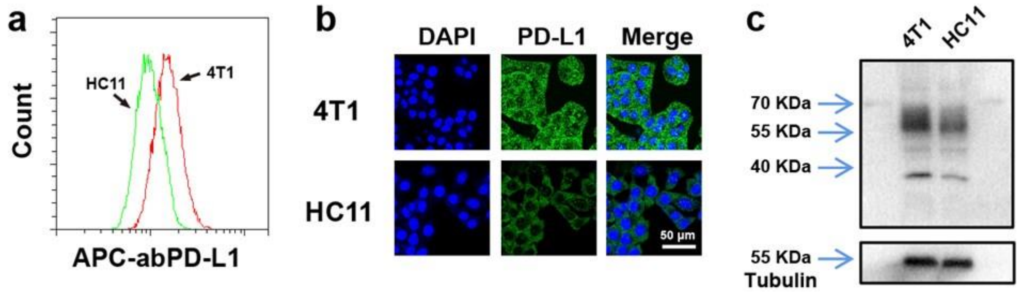

Supplementary Figure 10. Evaluation on the PD-L1 expression in TNBC cells. The PD-L1 expression in 4T1 cells and HC11 cells detected by flow cytometry (a), fluorescence confocal microscopy (b) and western blot (c). Flow cytometry, immunofluorescence and western blot experiments in panels a-c were repeated three times independently with similar results.

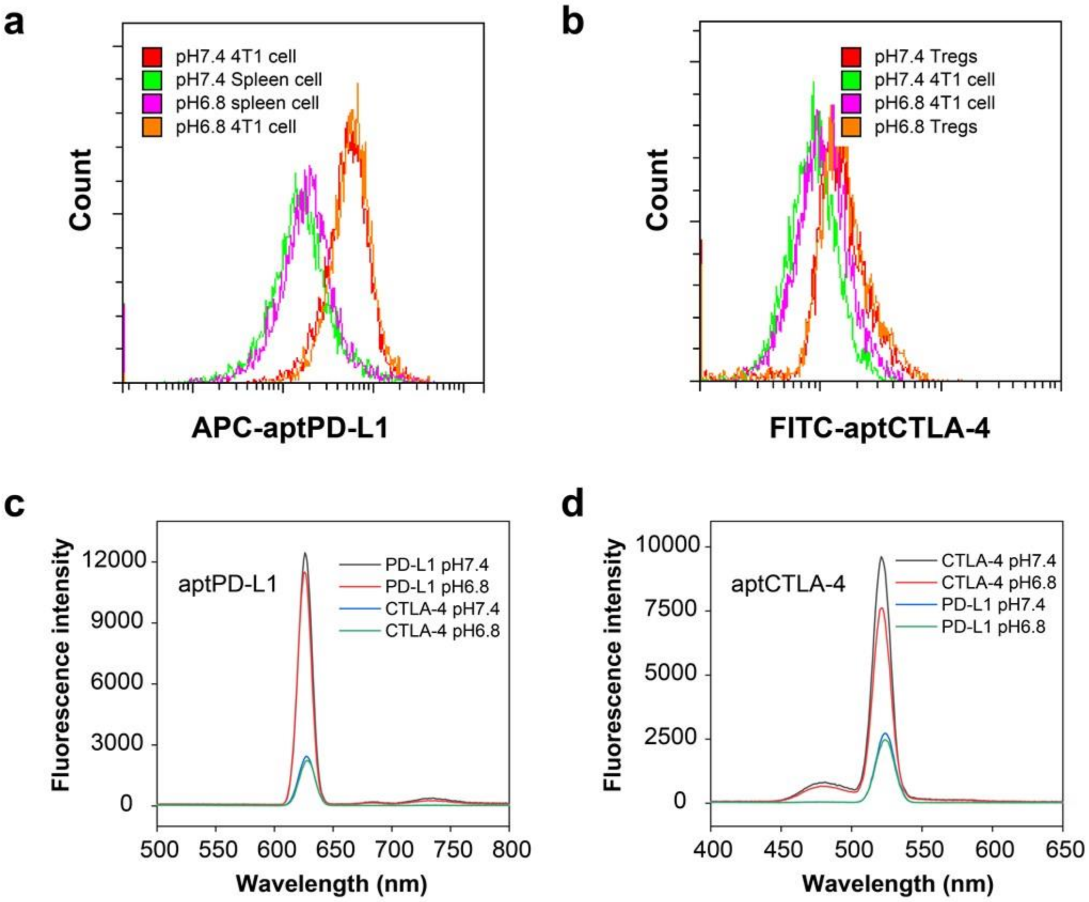

Supplementary Figure 11. Investigation of the impact of pH variations on the binding ability of aptPD-L1 and aptCTLA-4. (a-b) Cell binding behavior of aptPD-L1 and aptCTLA-4 in the co-incubation system of 4T1 cells and splenic immune cells under different pH conditions. (c-d) Evaluation on the binding of aptPD-L1/aptCTLA-4 to mouse PD-L1/CTLA-4 protein-modified magnetic beads under different pH conditions. Flow cytometry experiments in panels a-b were repeated three times independently with similar results. The fluorescence experiments in panels c-d were repeated three times independently with similar results. Source data are provided as a Source Data file.

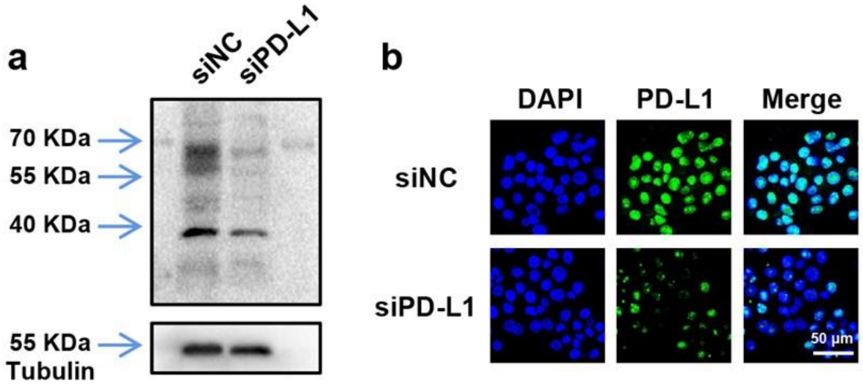

Supplementary Figure 12. Investigation of DNA-PAE endocytosis by 4T1 cells under neutral pH. (a) WB analysis on siPD-L1-mediated PD-L1 knock-down effect in 4T1 cells. (b) Endocytosis of DNA-PAE by 4T1 cells without/with PD-L1 knock-down. Scale bar = 50 μm. Western blot and immunofluorescence experiments in panels a-b were repeated three times independently with similar results.

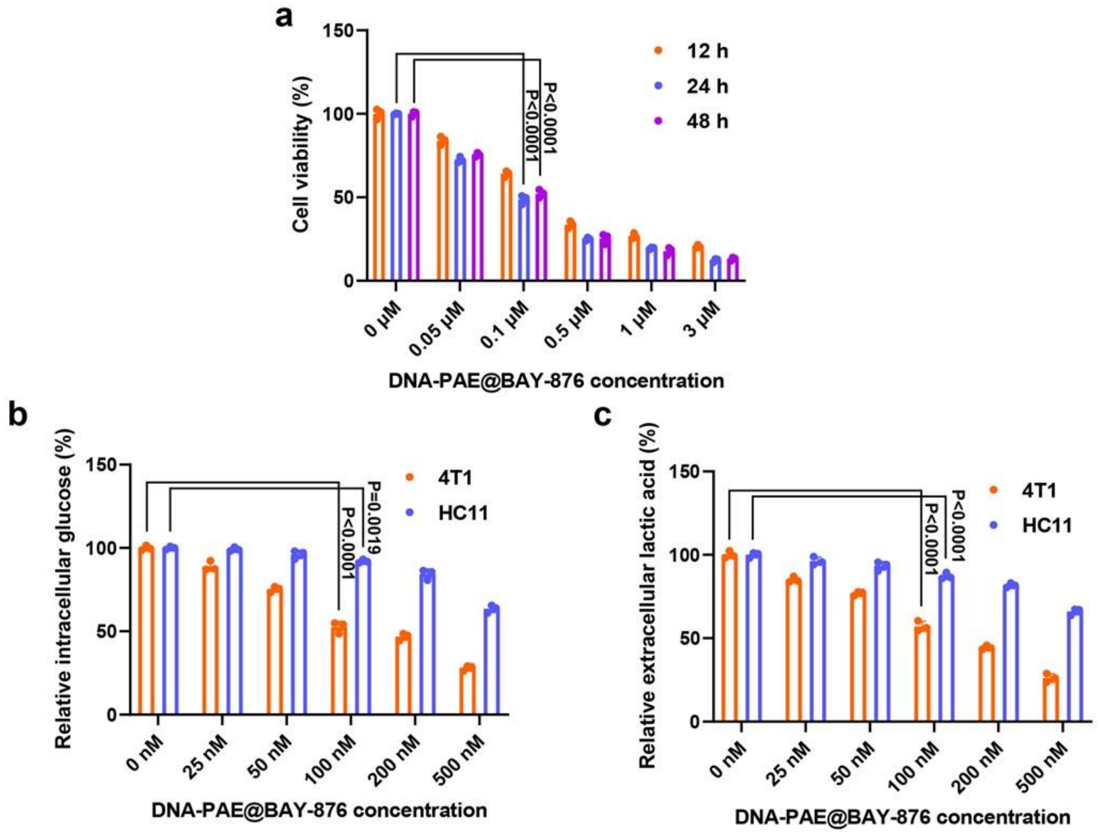

Supplementary Figure 13. Determination of the treatment conditions of DNA-PAE@BAY-876. (a) 4T1 cell killing effect by DNA-PAE@BAY-876 at different concentrations and different incubation times. (b-c) Glycolysis inhibition effects against 4T1 cells /HC11 cells by DNA-PAE@BAY-876 at different concentrations. Data are presented as mean values ± SEM (n = 3 biologically independent samples for panels a-c). Statistical analysis was carried out via two-way ANOVA method. Source data are provided as a Source Data file.

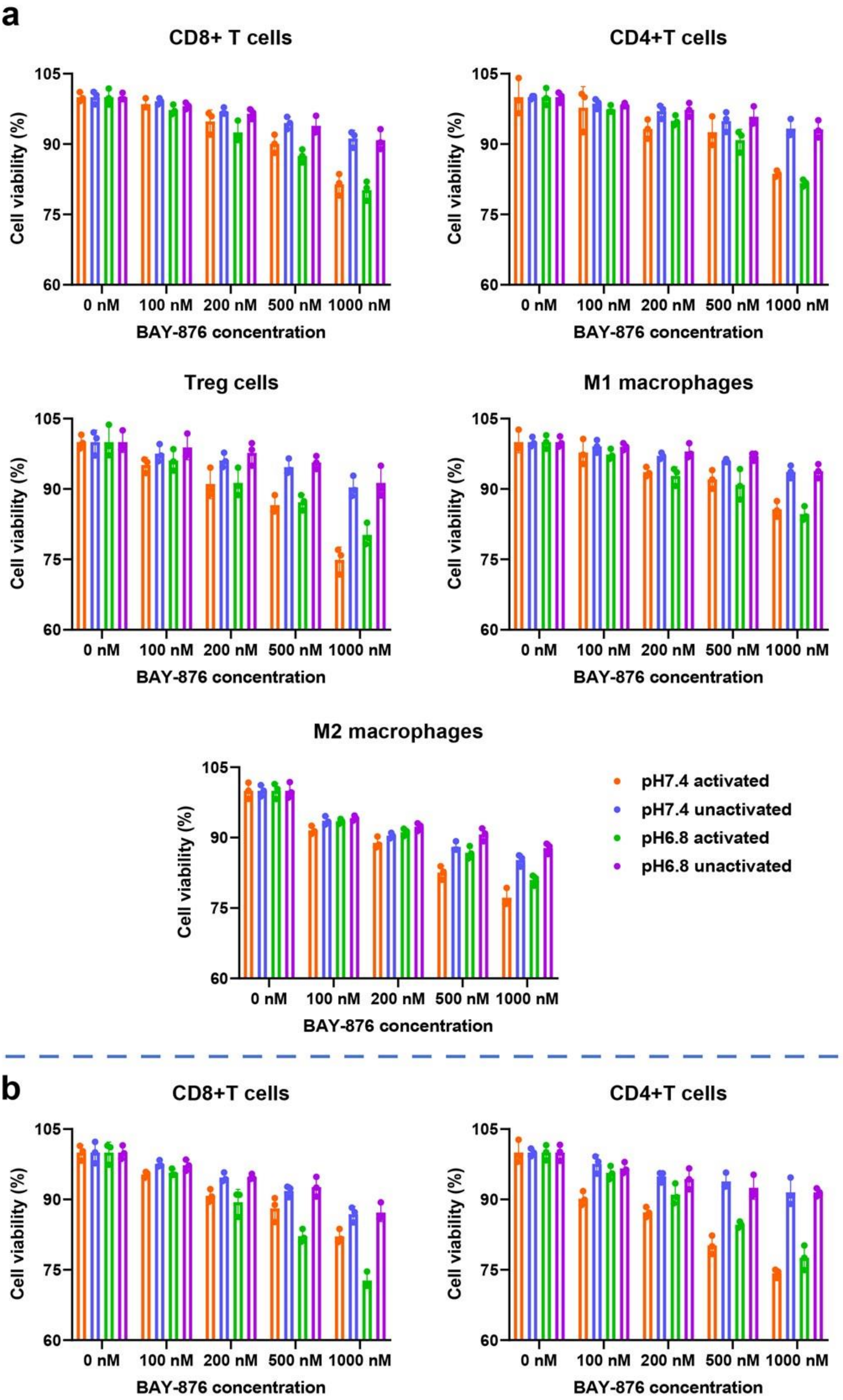
a
CD8+ T cells
CD4+T cells
Treg cells
M1 macrophages
M2 macrophages
Cell viability (%)
105
90
75
60
0 nM
100 nM
200 nM
500 nM
1000 nM
BAY-876 concentration
pH7.4 activated
pH7.4 unactivated
pH6.8 activated
pH6.8 unactivated
b
CD8+T cells
CD4+T cells

Supplementary Figure 14. Concentration-dependent impact of BAY-876 to major immune cell populations under the activation period of 48 h (a) or 96 h (b). Data are presented as mean values ± SEM (n = 3 biologically independent samples for panels a-b). Source data are provided as a Source Data file.

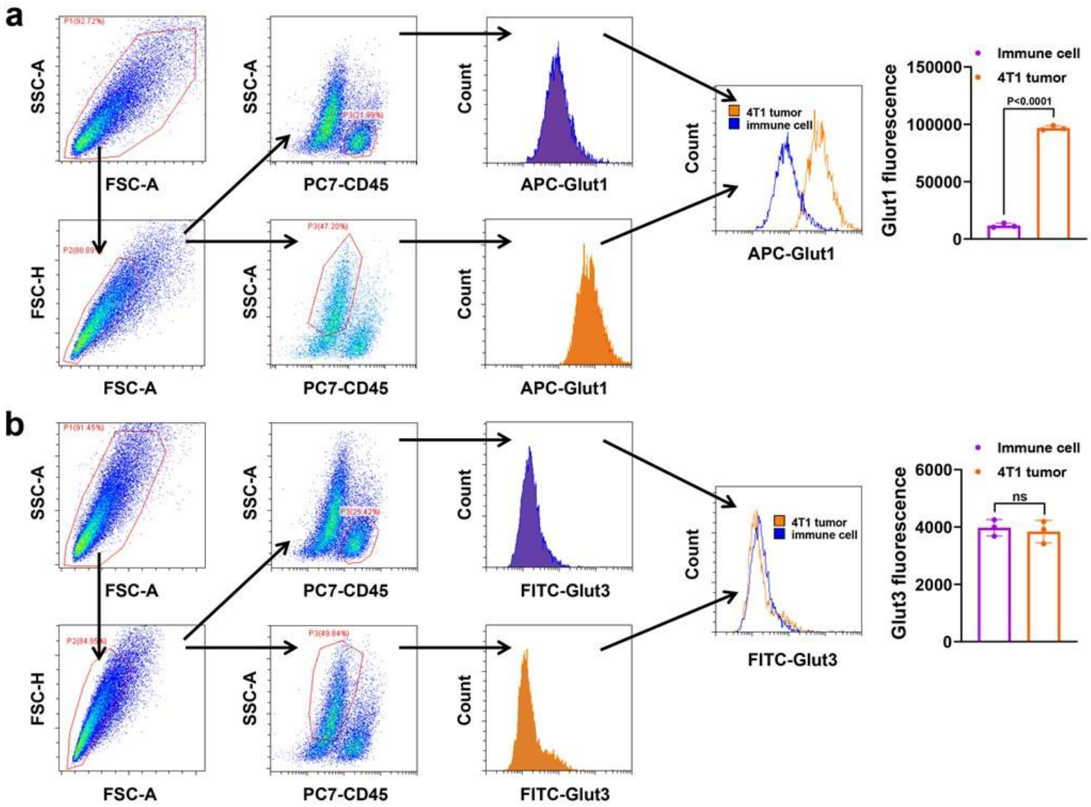

Supplementary Figure 15. GLUT1 (a) and GLUT3 (b) distribution in different cell populations in vivo by flow cytometry. Flow cytometry experiments in panels a-b were repeated three times independently with similar results. Data are presented as mean values ± SEM (n = 3 biologically independent samples for panels a and b). Statistical analysis was carried out via one-way ANOVA method. Source data are provided as a Source Data file.

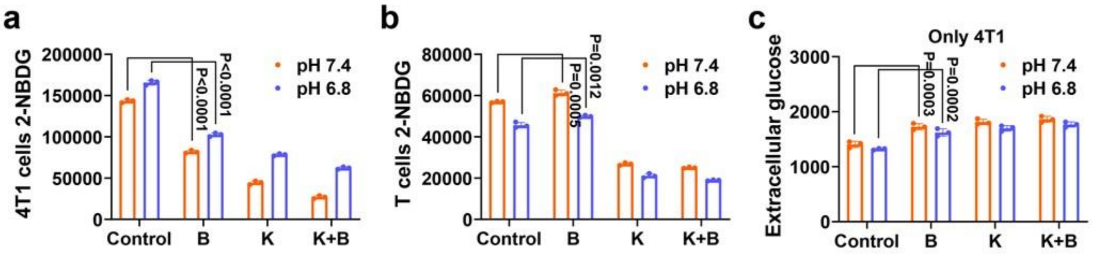

Supplementary Figure 16. Glucose uptake rate in cells after BAY-876 or KL-11743 treatment. (a-b) Impact of different GLUT inhibitors on 2-NBDG uptake in 4T1 cells or T cells in the co-culture system (n=3). (c) Extracellular glucose levels in 4T1 cell incubation media after treatment by BAY-876 or KL-11743 to 4T1 cells alone by 2-NBDG. Data are presented as mean values ± SEM (n = 3 biologically independent samples for panels a-c). Statistical analysis was carried out via two-way ANOVA method. Source data are provided as a Source Data file.

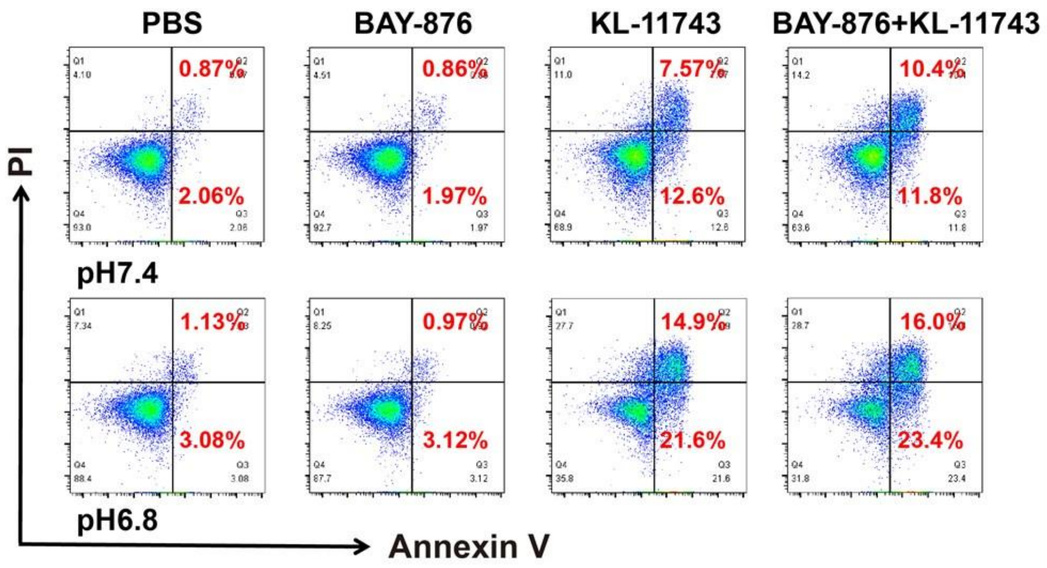

Supplementary Figure 17. Apoptosis of T cells in the co-incubation system of 4T1 cells and T cells after treatment with BAY-876 or KL-11743. Flow cytometry experiments were repeated three times independently with similar results.

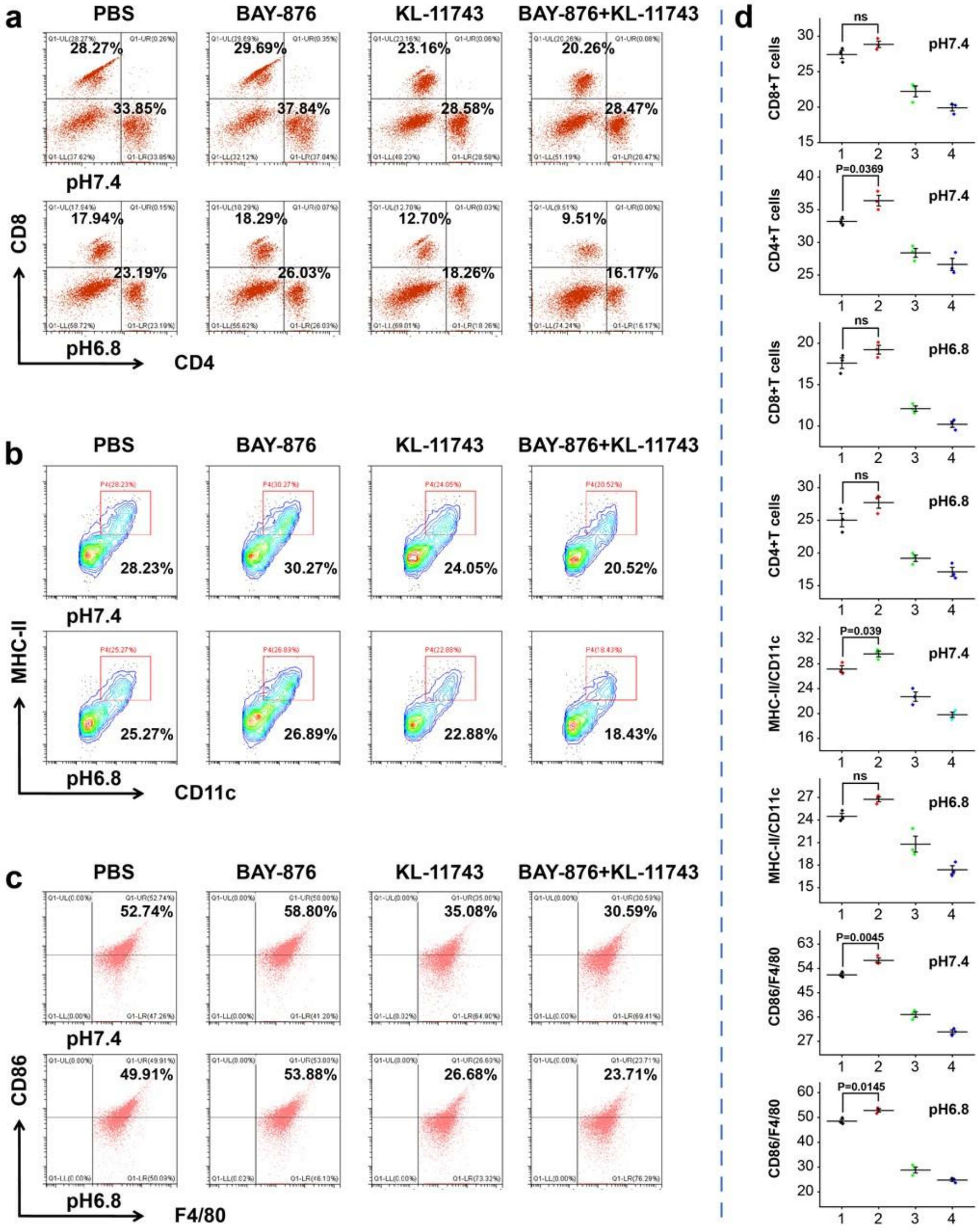

Supplementary Figure 18. Phenotypical changes of various immune cells in the co-incubation system of 4T1 cells and splenocytes after BAY-876 or KL-11743 treatment. (a) Flow cytometry evaluation on the expansion of CD8+/CD4+ T cells. (b) Flow cytometry evaluation of DC maturation (CD11c+MHC-II+). (c) Flow cytometry evaluation on the polarization state of macrophages (F4/80+CD86+). (d) Statistical

analysis of flow cytometry data in panels a-c. (1) PBS, (2) BAY-876, (3) KL-11743, (4) BAY-876+KL-11743. Flow cytometry experiments in panels a-c were repeated three times independently with similar results. Data are presented as mean values ± SEM (n = 3 biologically independent samples for panel d). Statistical analysis was carried out via two-way ANOVA method. Source data are provided as a Source Data file.

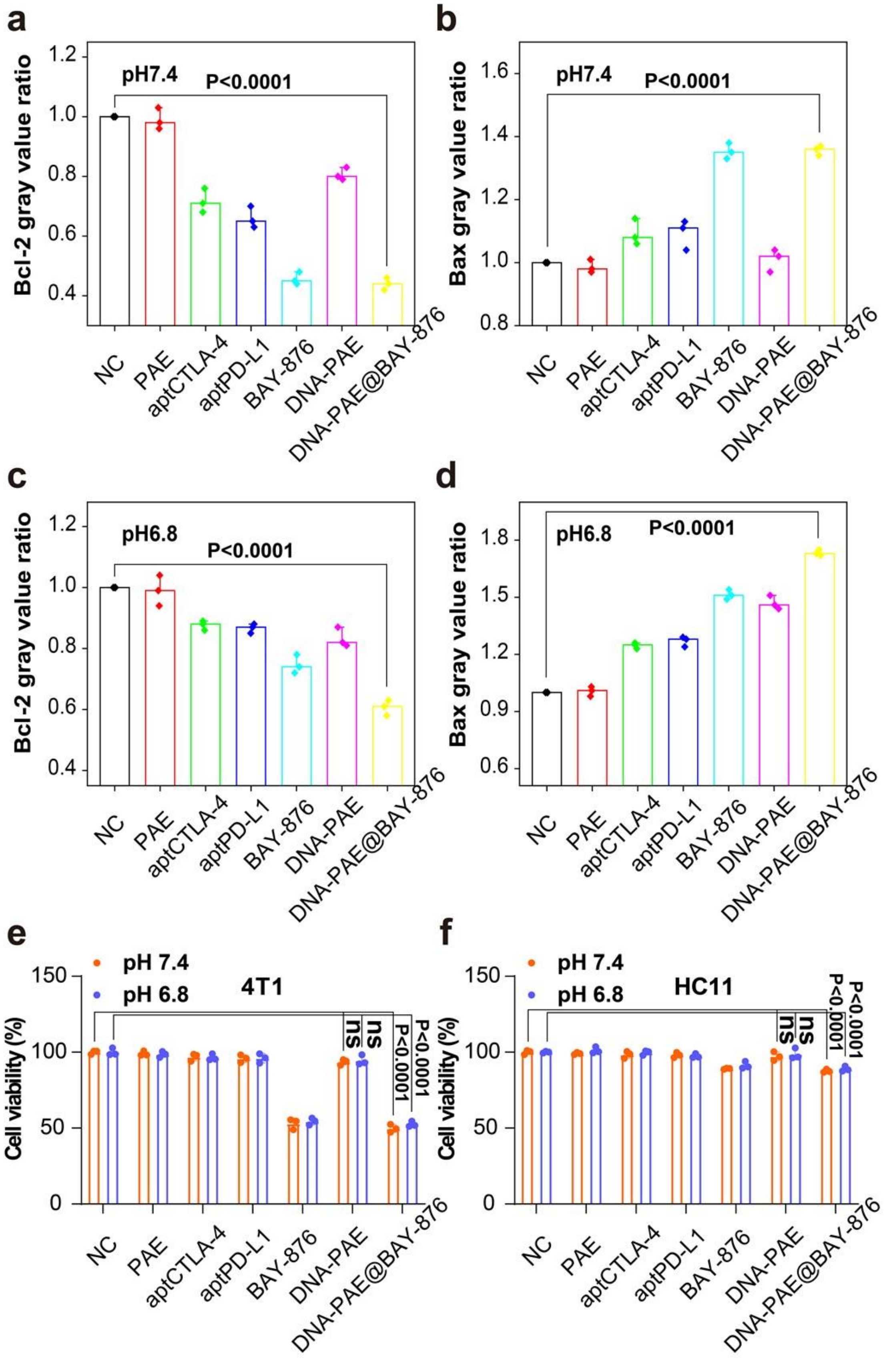

Supplementary Figure 19. Biochemical changes and survival of 4T1 cells after different

treatment. (a-d) Statistic analysis on the expression levels of key apoptosis-related markers in 4T1 cells after different treatment via WB assay. (e-f) Survival rate of 4T1 cells and HC11 cells after different treatment. Data are presented as mean values ± SEM (n = 3 biologically independent samples for panels a-f). Statistical analysis was carried out via two-way ANOVA method. Source data are provided as a Source Data file.

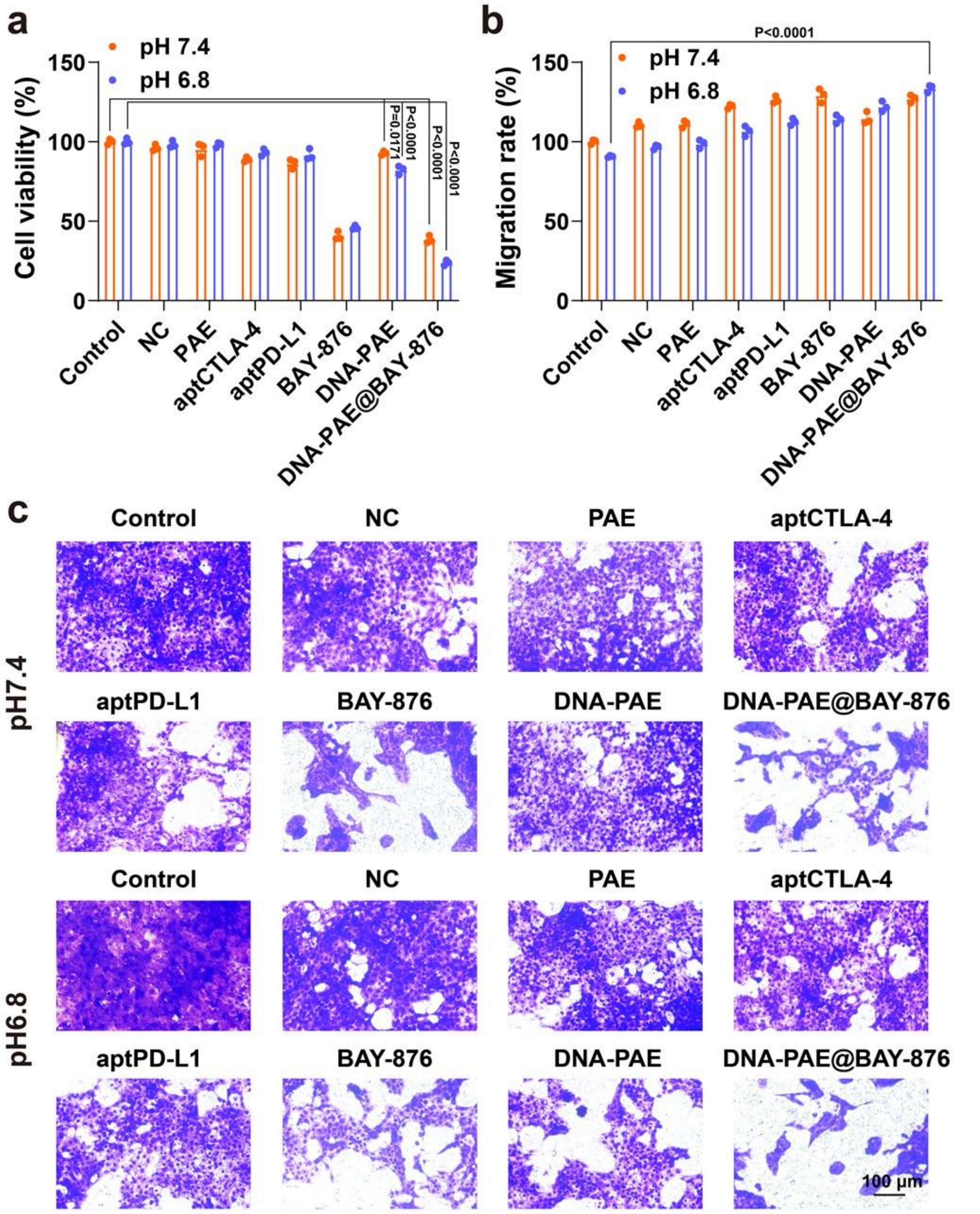

Supplementary Figure 20. Characterization of the 4T1 cell survival after different treatment. (a) 4T1 survival rate in the 4T1-splenic cell co-incubation system after different treatment. (b) Migration of T cells in the 4T1-splenic cell co-incubation system after various treatment. (c) Invasion capacity of 4T1 cells in the 4T1-splenic cell co-incubation system after different treatment. Invasion experiments in panel c were

repeated three times independently with similar results. Data are presented as mean values ± SEM (n = 3 biologically independent samples for panels a and b). Statistical analysis was carried out via two-way ANOVA method. Source data are provided as a Source Data file.

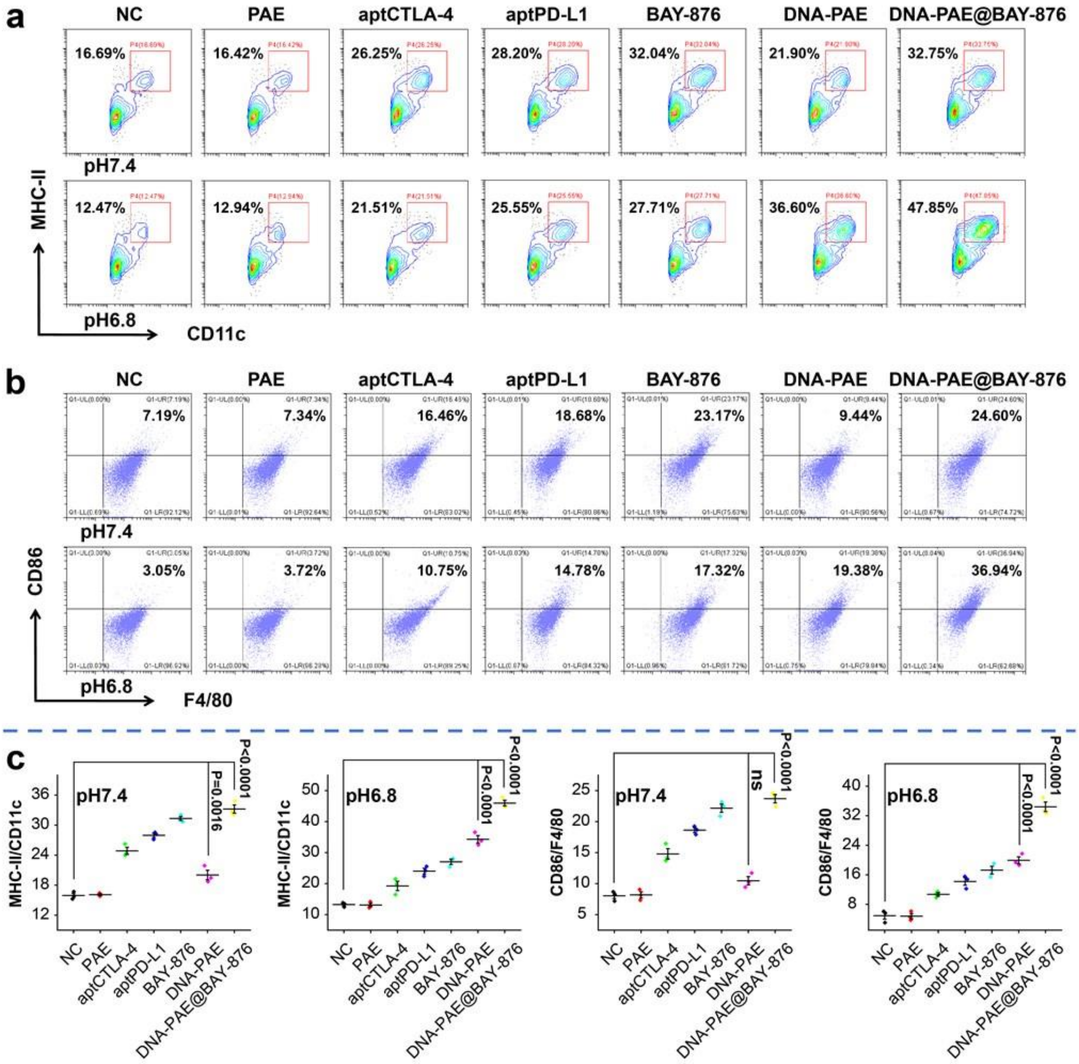

Supplementary Figure 21. Nanoassembly treatment boosted the activation of APCs. Flow cytometry evaluation of DC maturation (CD11c+MHC-II+) (a) and M1 polarization of macrophages (F4/80+CD86+) after various treatment. (b) in the co-incubation system after different treatment. (c) Statistical analysis of flow cytometry data in panels a-b. Flow cytometry experiments in panels a-b were repeated three times independently with similar results. Data are presented as mean values ± SEM (n = 3 biologically independent samples for panel c). Statistical analysis was carried out via two-way ANOVA method. Source data are provided as a Source Data file.

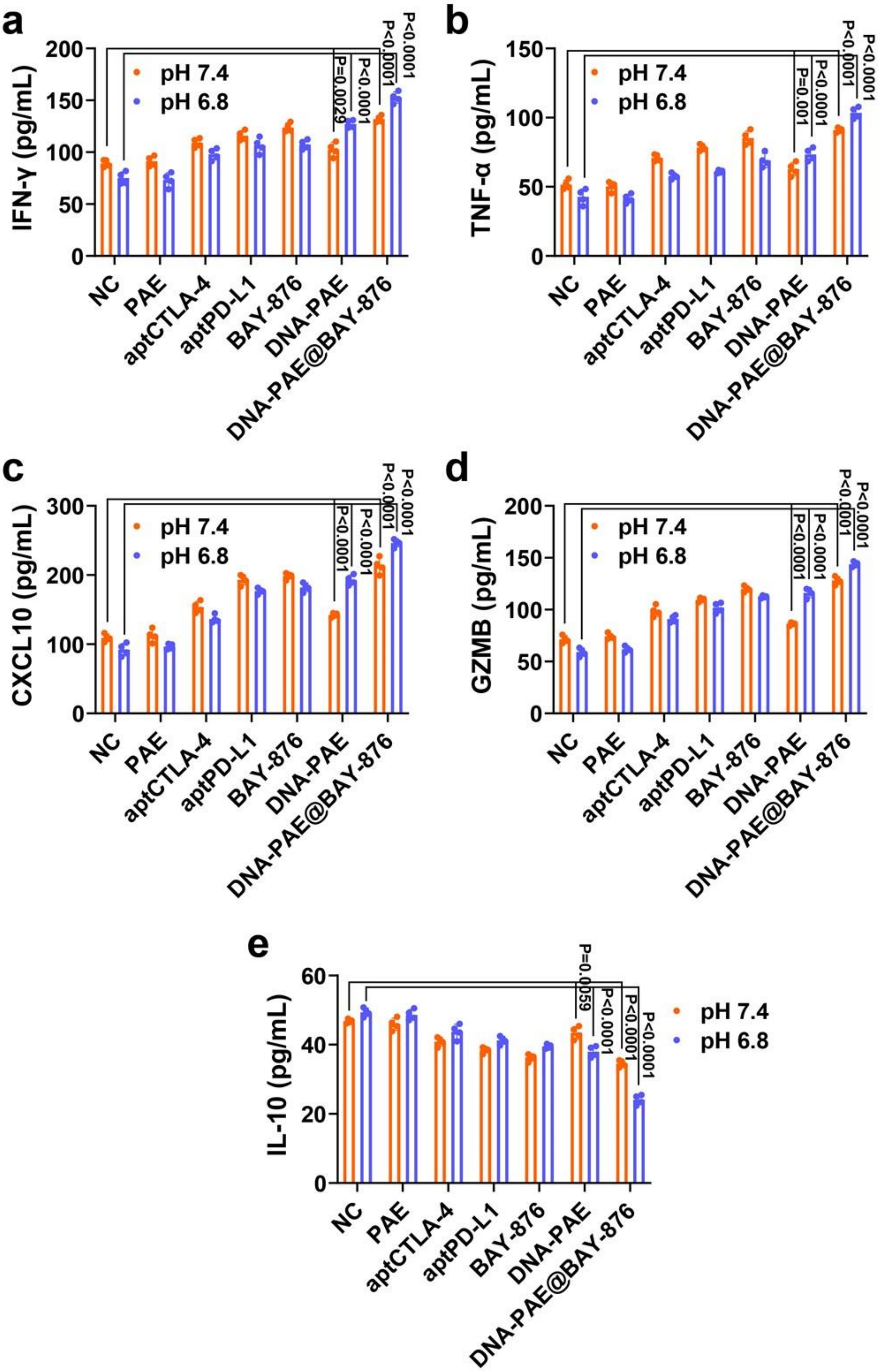

Supplementary Figure 22. ELISA assay on the secretion of key immune-related cytokines and chemokines in the co-culture system after different treatment. Data are

presented as mean values ± SEM (n = 4 biologically independent samples for panels a-e). Statistical analysis was carried out via two-way ANOVA method. Source data are provided as a Source Data file.

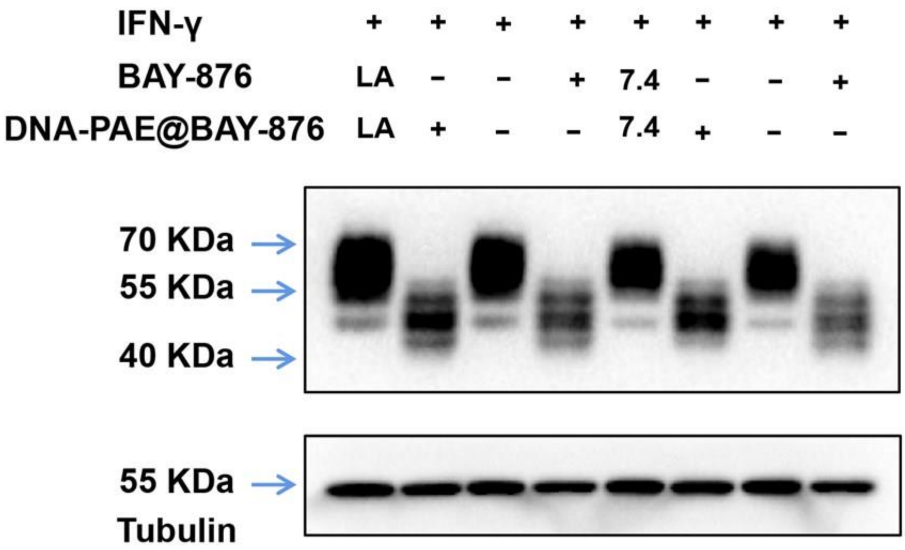

Supplementary Figure 23. PD-L1 glycosylation of 4T1 cells after treatment with 100 nM BAY-876/DNA-PAE@BAY-876. Note: the molecular weight of PD-L1 is 33-70 KDa; the molecular weight of Tubulin is 50 KDa. Western blot experiments were repeated three times independently with similar results.

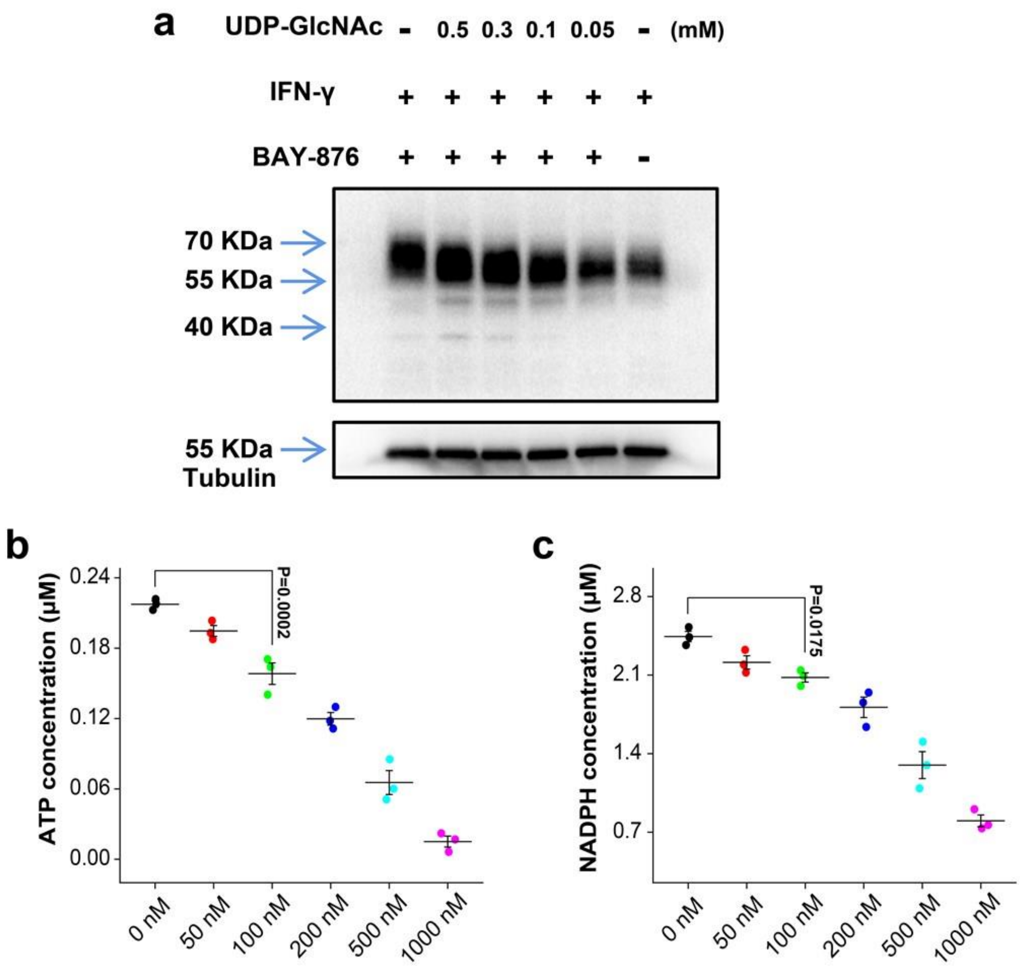

Supplementary Figure 24. Effects of BAY-876 or UDP-GlcNAc with different concentrations to 4T1 cells. (a) Effect of UDP-GlcNAc with different concentrations to the PD-L1 glycosylation on 4T1 cells treated with 100 nM BAY-876. Note: the molecular weight of PD-L1 is 33-70 KDa; the molecular weight of Tubulin is 50 KDa. (b) Changes of ATP content in 4T1 cells by BAY-876 with different concentrations. (c) Changes of NADPH content in 4T1 cells by BAY-876 with different concentrations. Western blot experiments in panel a were repeated three times independently with similar results. Data are presented as mean values ± SEM (n = 3 biologically independent samples for panels b and c). Statistical analysis was carried out via two-way ANOVA method. Source data are provided as a Source Data file.

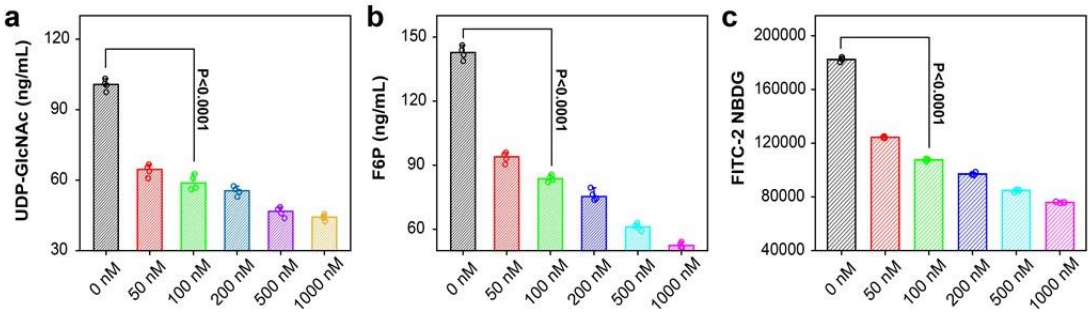

Supplementary Figure 25. Changes in the abundance of key glycolysis products after treatment with BAY-876 at different concentrations. (a) UDP-GlcNAc; (b) F6P; (c) 2-NBDG. Data are presented as mean values ± SEM (n = 4 biologically independent samples for panels a and b, n = 3 biologically independent samples for panel c). Statistical analysis was carried out via two-way ANOVA method. Source data are provided as a Source Data file.

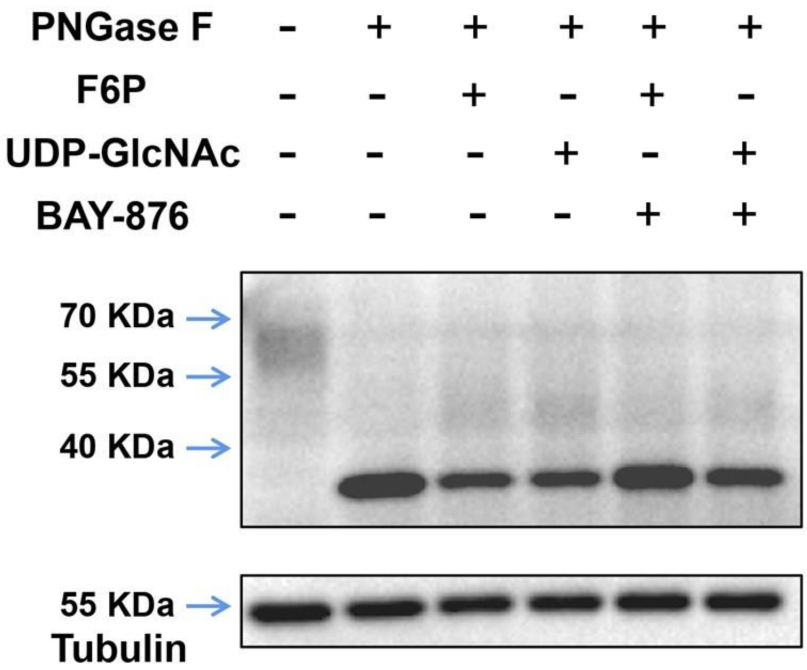

Supplementary Figure 26. PD-L1 deglycosylation of 4T1 cells after PNGase F treatment. Note: the molecular weight of PD-L1 is 33-70 KDa; the molecular weight of Tubulin is 50 KDa. Western blot experiments were repeated three times independently with similar results.

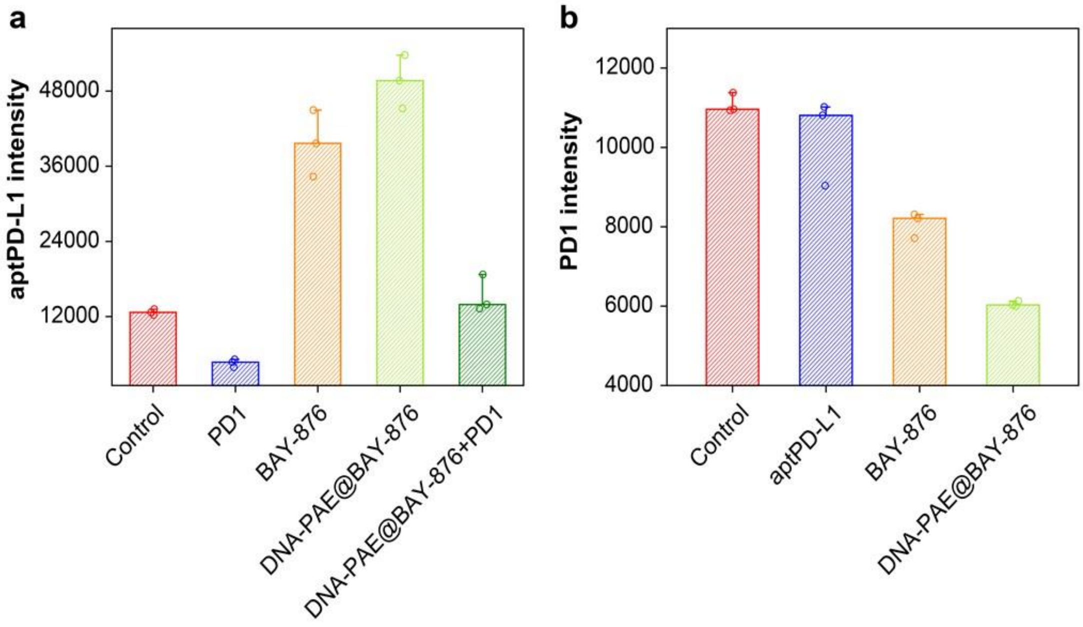

Supplementary Figure 27. Quantitative analysis on the competitive binding between aptPD-L1 and PD-1 with PD-L1 on 4T1 cell surface via flow cytometry. Data are presented as mean values ± SEM (n = 3 biologically independent samples for panels a and b). Statistical analysis was carried out via two-way ANOVA method. Source data are provided as a Source Data file.

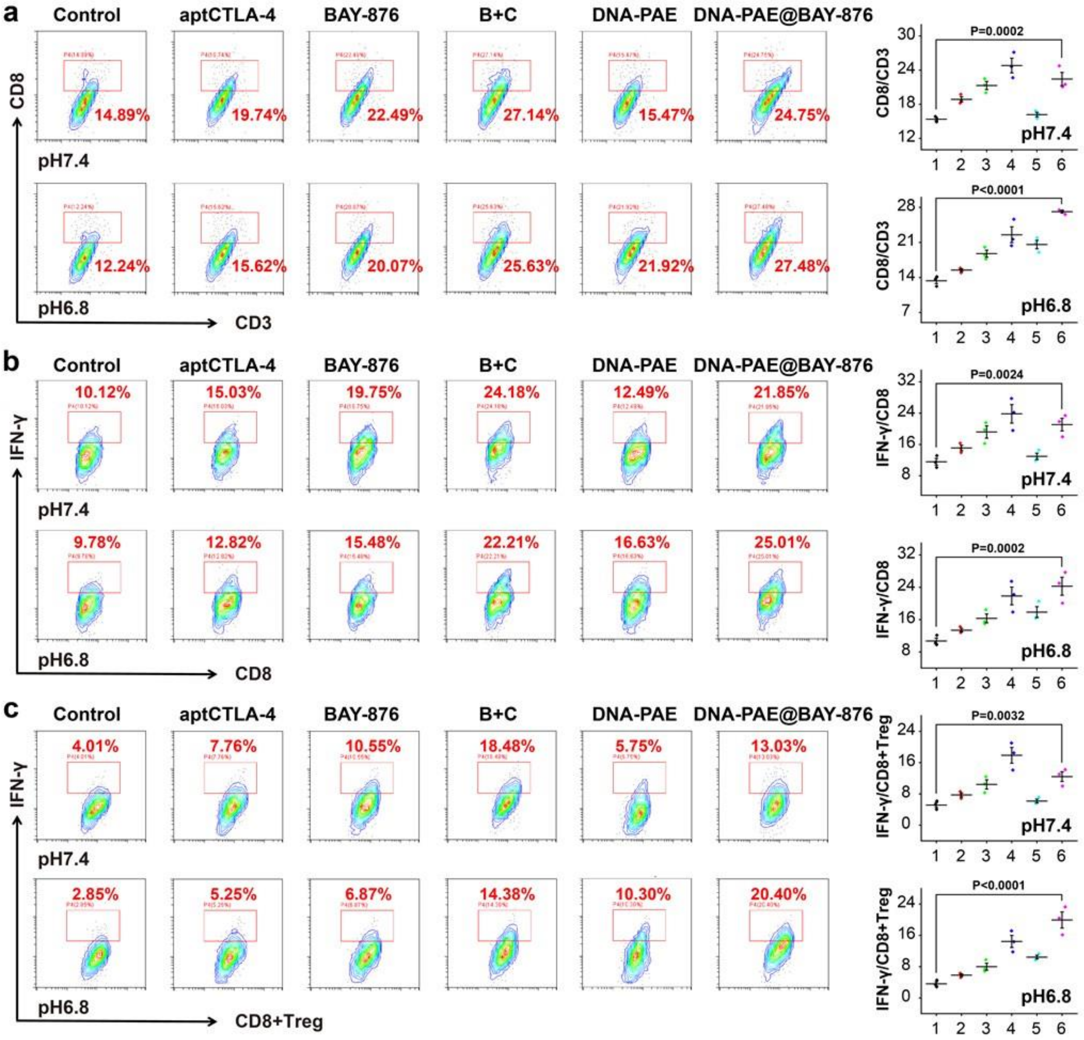

Supplementary Figure 28. Phenotypical changes of CD8+T cells under different co-culture conditions with different treatment. (a) Frequency of CD8+ T cells in the co-incubation system of 4T1 cells, CD8+ T cells and Tregs after different treatment. (b) Frequency of IFN-γ+ CD8+ T cells in the co-incubation system of 4T1 cells and CD8+ T cells after different treatment. (c) Frequency of IFN-γ+ CD8+ T cells in the co-incubation system of 4T1 cells, Tregs and CD8+ T cells after different treatment. (1) Control, (2) aptCTLA-4, (3) BAY-876, (4) B+C, (5) DNA-PAE, (6) DNA-PAE@BAY-876. B+C was BAY-876 combined with aptCTLA-4. Flow cytometry experiments in panels a-c were repeated three times independently with similar results. Data are

presented as mean values ± SEM (n = 3 biologically independent samples for panels a-c). Statistical analysis was carried out via two-way ANOVA method. Source data are provided as a Source Data file.

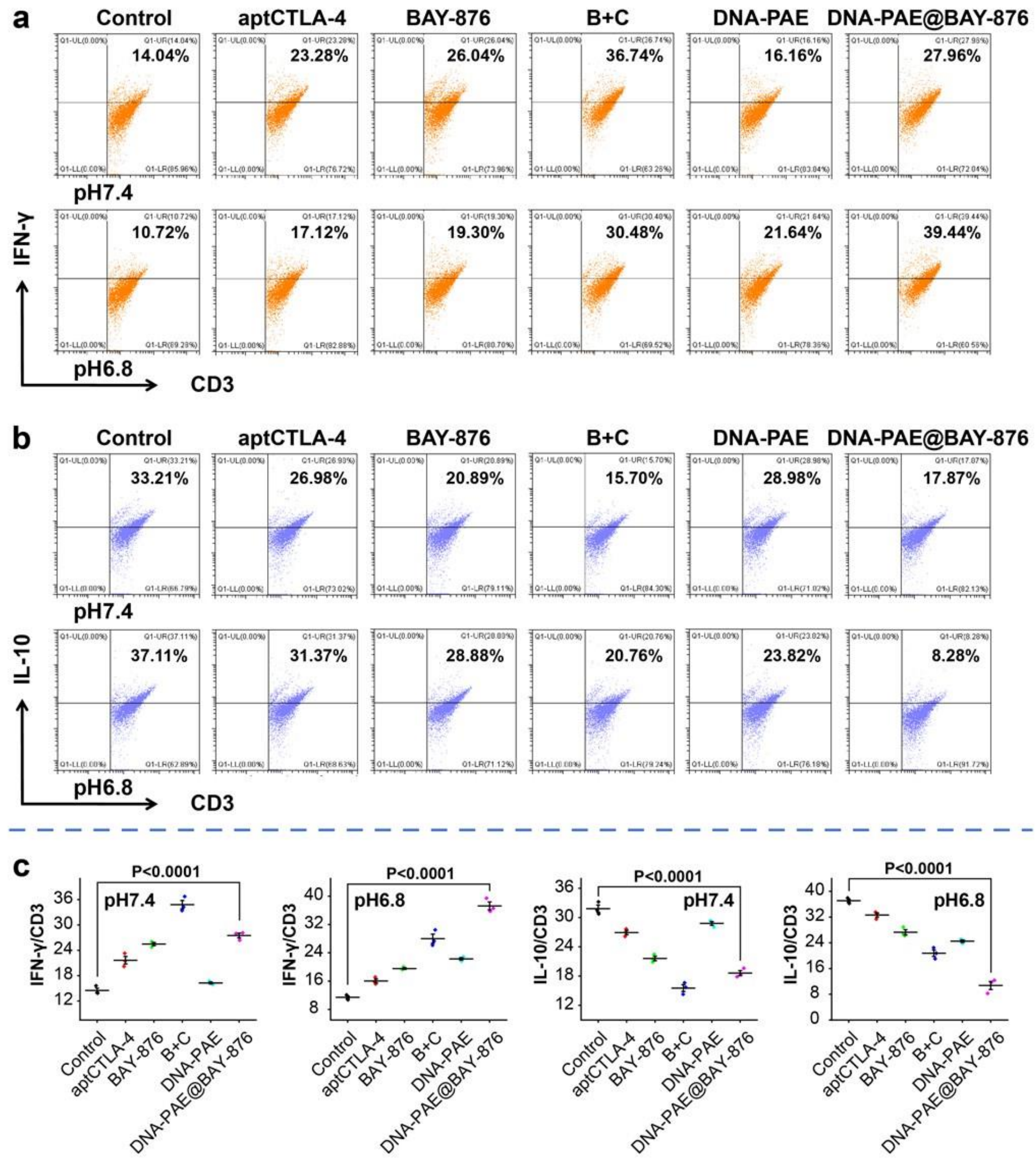

Supplementary Figure 29. Activation status of T cells in the co-incubation system of 4T1 cells and spleen cells after different treatment. (a) IFN-γ secretion in the co-incubation system of 4T1 cells and spleen cells after different treatment. (b) IL-10 secretion in the co-incubation system of 4T1 cells and spleen cells after different treatment. (c) Statistical analysis the flow cytometric data in panels a-b. B+C means BAY-876 combined with aptCTLA-4. Flow cytometry experiments in panels a and b were repeated three times independently with similar results. Data are presented as

mean values ± SEM (n = 3 biologically independent samples for panel c). Statistical analysis was carried out via two-way ANOVA method. Source data are provided as a Source Data file.

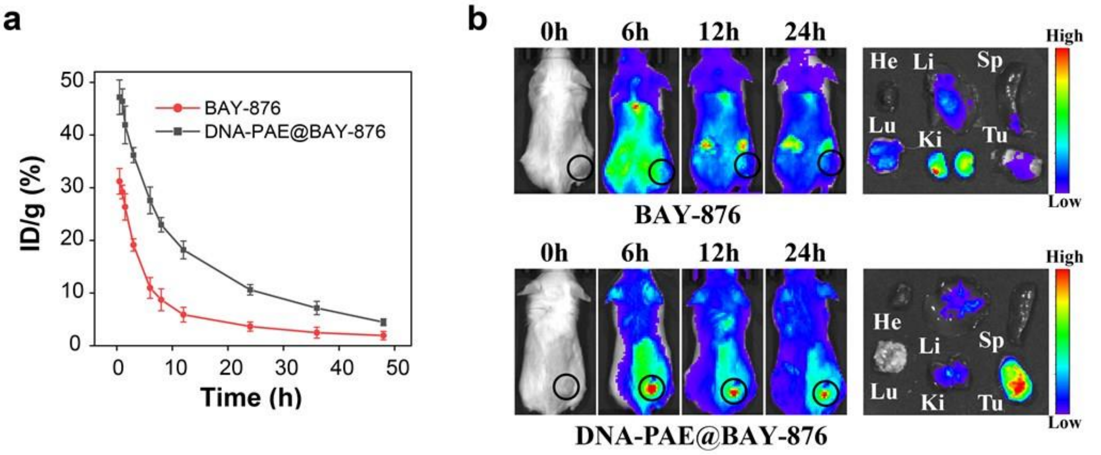

Supplementary Figure 30. In vivo pharmacokinetic analysis. (a) Pharmacokinetic profiles of DNA-PAE@BAY-876 and BAY-876 in mice after intravenous injection. (b) Systemic distribution of DNA-PAE@BAY-876/BAY-876 at 0/6/12/24h post intravenous injection with three mice per group. In vivo imaging experiments in panel b were repeated three times independently with similar results. Data are presented as mean values ± SEM (n = 3 mice for panel a). Source data are provided as a Source Data file.

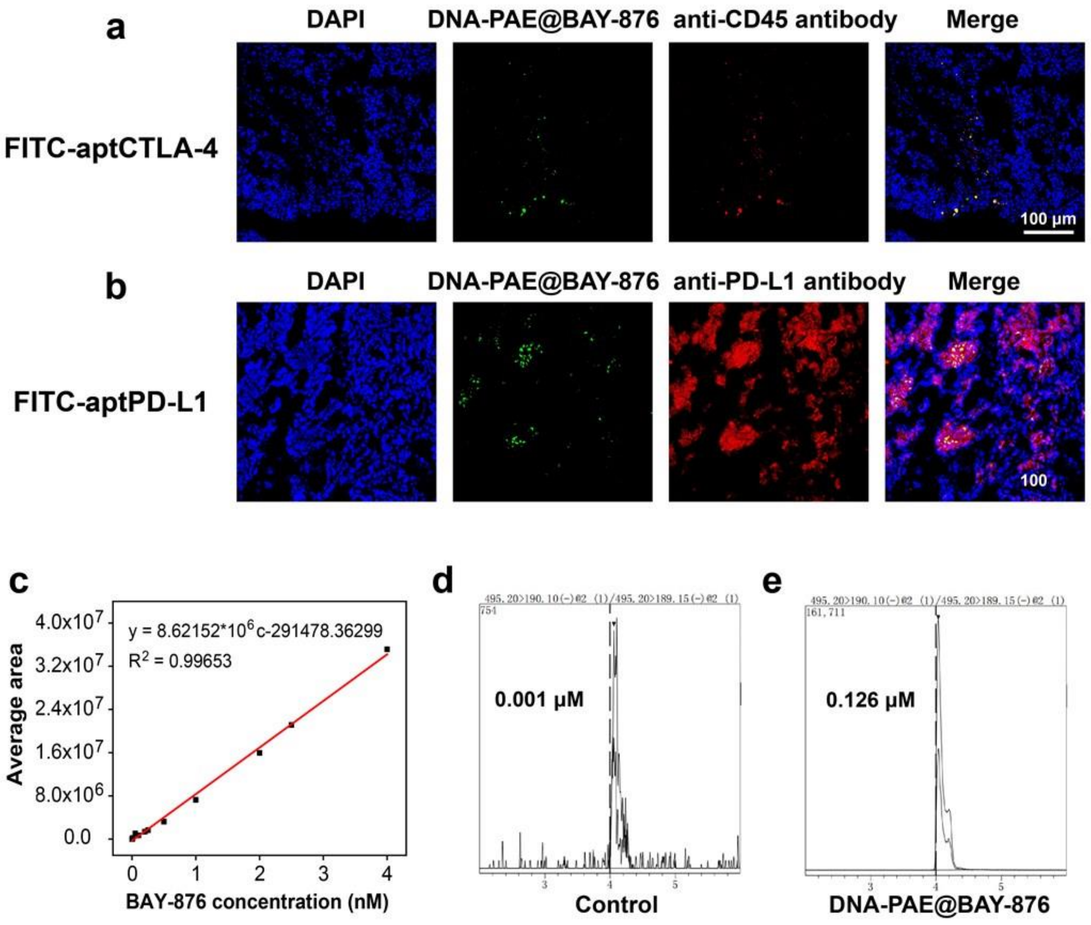

Supplementary Figure 31. The distribution of DNA-PAE@BAY-876 in the tumor of 4T1 tumor-bearing mice. (a-b) The distribution of aptCTLA-4 and aptPD-L1 in vivo with three mice per group. (c-e) The quantification of BAY-876 content in the tumor by liquid chromatography-triple quadrupole mass spectrometer. Immunofluorescence experiments in panels a-b were repeated three times independently with similar results. Source data are provided as a Source Data file.

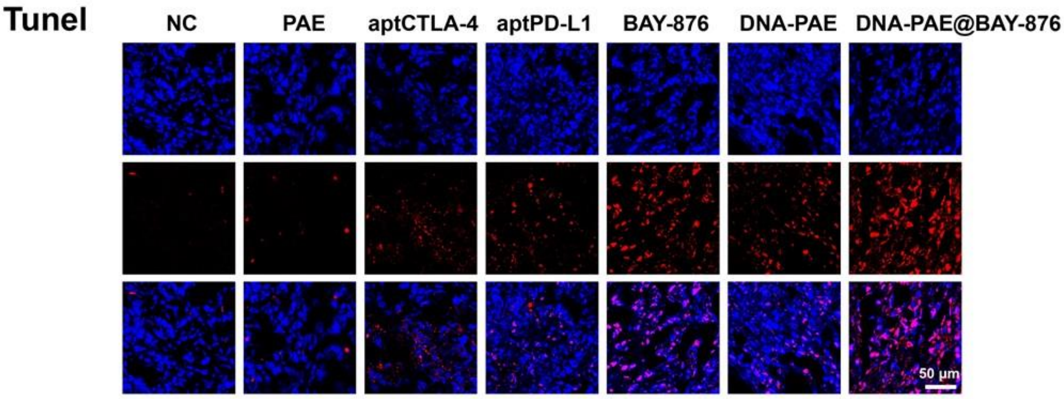

Supplementary Figure 32. Confocal images of 4T1 cell apoptosis in vivo after 21 days under different treatments with five mice per group. Immunofluorescence experiments were repeated three times independently with similar results.

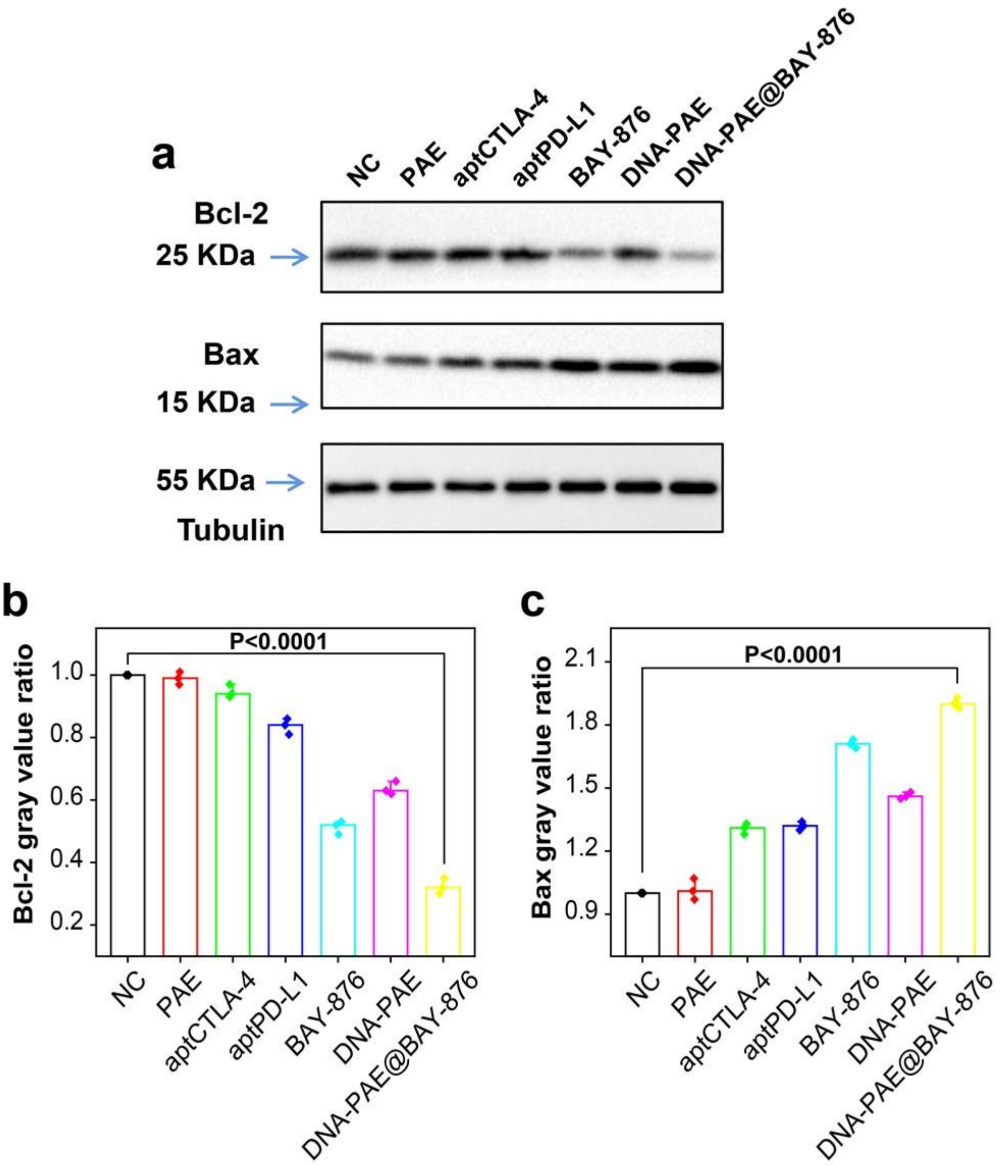

Supplementary Figure 33. Apoptosis effect of 4T1 tumor cells in vivo after 21 days of different treatment. (a) Apoptosis effect of 4T1 tumor by western blotting. (b-c) Apoptosis gray value statistics by western blotting. Note: the molecular weight of Bcl-2 is 26 KDa; the molecular weight of Bax is 21 KDa; the molecular weight of Tubulin is 50 KDa. In vivo western blot experiments in panel a were repeated three times independently with similar results. Data are presented as mean values ± SEM (n = 3 mice for panels b-c). Statistical analysis was carried out via two-way ANOVA method. Source data are provided as a Source Data file.

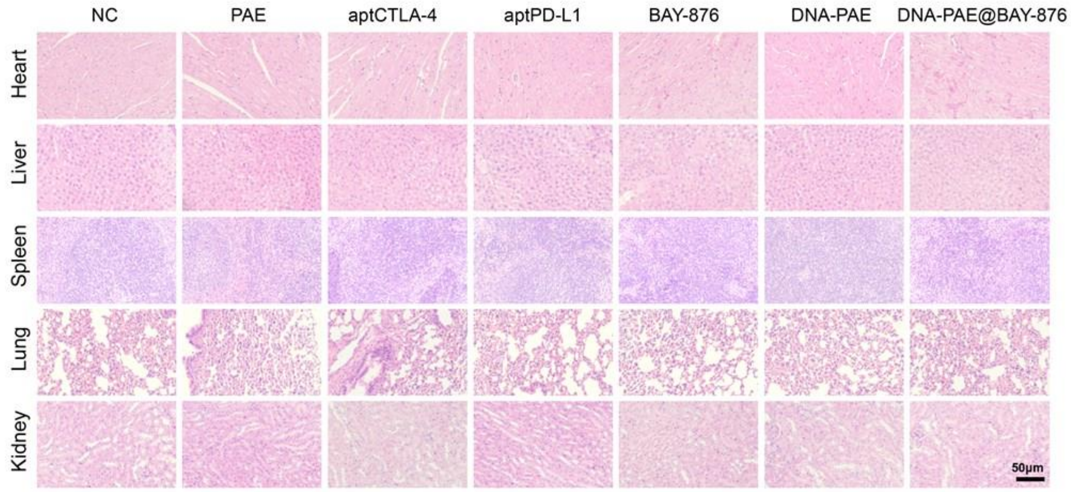

Supplementary Figure 34. Histocompatibility of the nanoassembly. H&E staining was performed on major organ tissue samples of 4T1 tumor-bearing mice after different treatments with five mice per group. In vivo histological experiments were repeated three times independently with similar results.

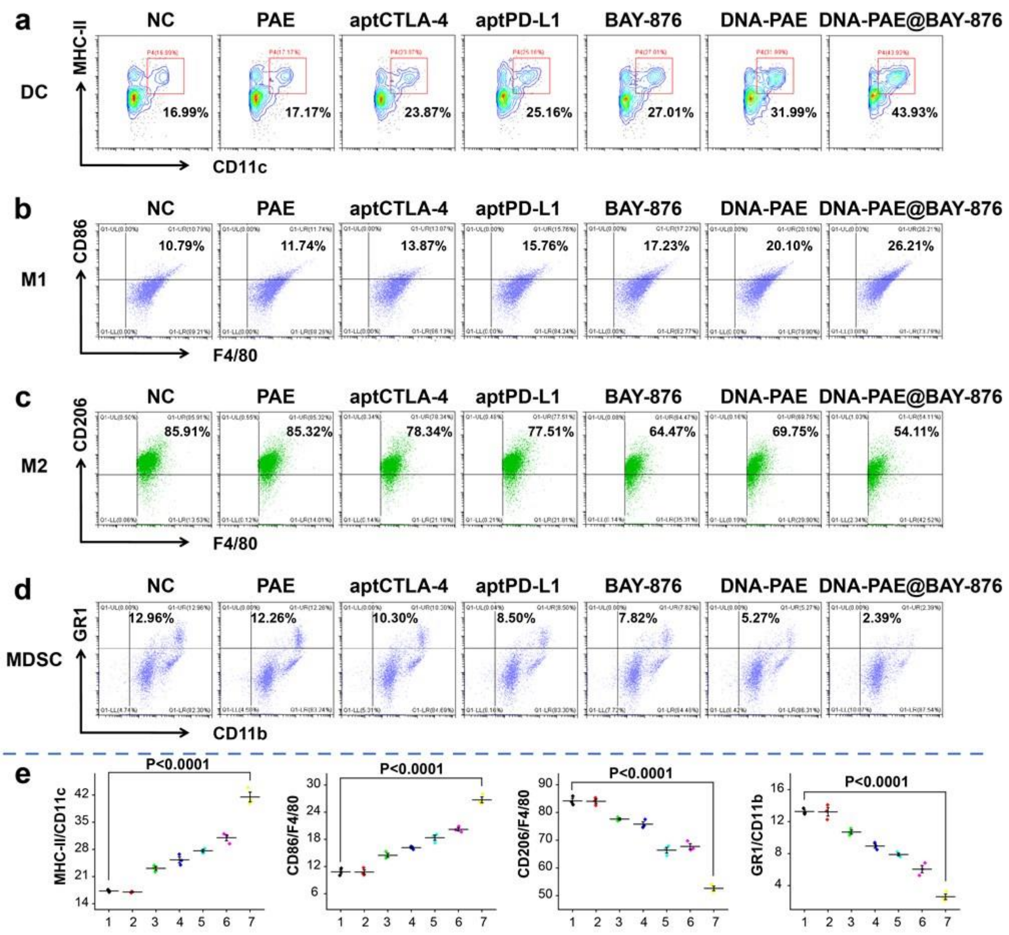

Supplementary Figure 35. Evaluation on DNA-PAE@BAY-876-mediated immunotherapy in 4T1 tumor-bearing mice. (a) Flow cytometry detection on the tumor infiltration of DCs (CD11c+MHC-II+). (b) Flow cytometry detection on the tumor-infiltration of M1 macrophages (F4/80+CD86+). (c) Flow cytometry detection on the tumor infiltration of M2 macrophages (F4/80+CD206+). (d) Flow cytometry detection on the tumor infiltration of MDSCs (CD11b+GR1+). (e) Statistical analysis of a-d data by flow cytometry. (1) NC, (2) PAE, (3) aptCTLA-4, (4) aptPD-L1, (5) BAY-876, (6) DNA-PAE, (7) DNA-PAE@BAY-876. Flow cytometry experiments in panels a-d were repeated three times independently with similar results. Data are presented as mean values ± SEM (n = 3 mice for panel e). Statistical analysis was carried out via two-way ANOVA method. Source data are provided as a Source Data file.

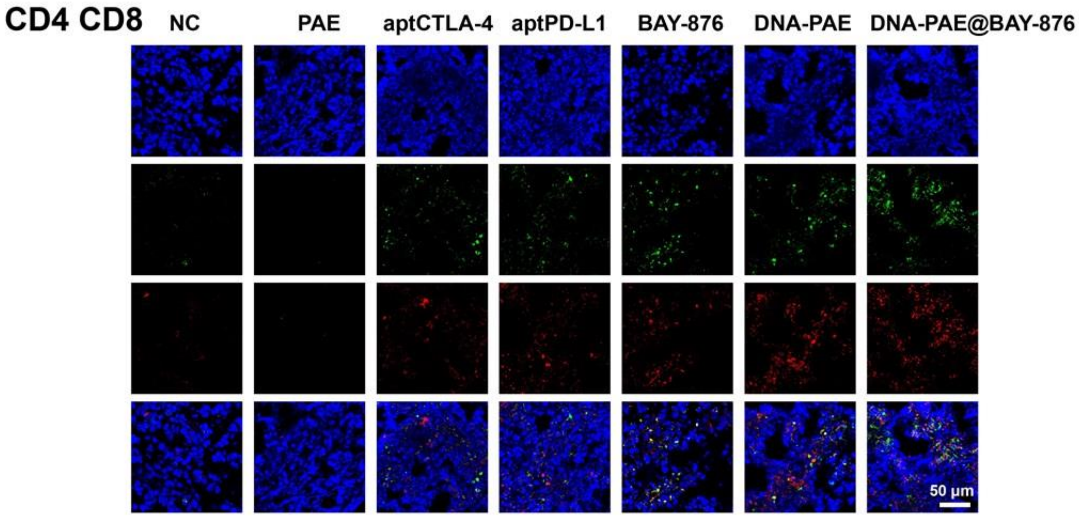

Supplementary Figure 36. Infiltration of CD4+ T cells and CD8+ T cells in 4T1 tumors after 21 days of different treatment with five mice per group. Immunofluorescence experiments were repeated three times independently with similar results.

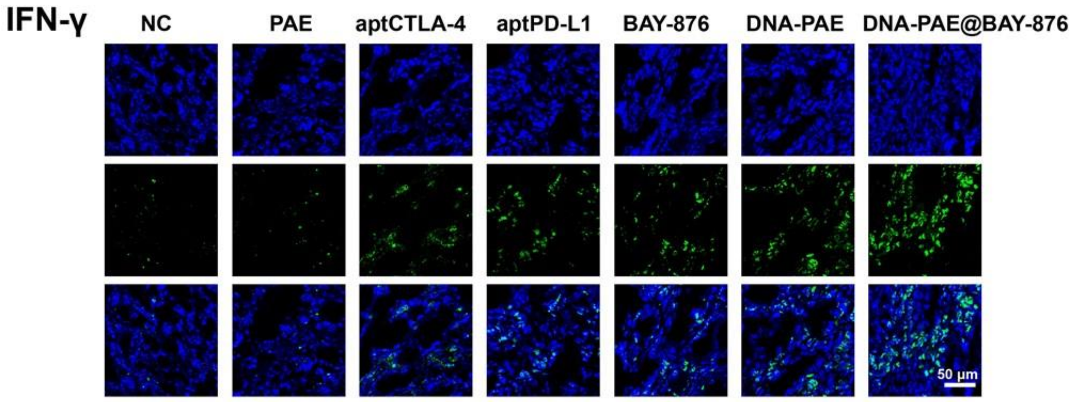

Supplementary Figure 37. IFN-γ secretion levels in 4T1 tumors after 21 days of different treatments with five mice per group. Immunofluorescence experiments were repeated three times independently with similar results.

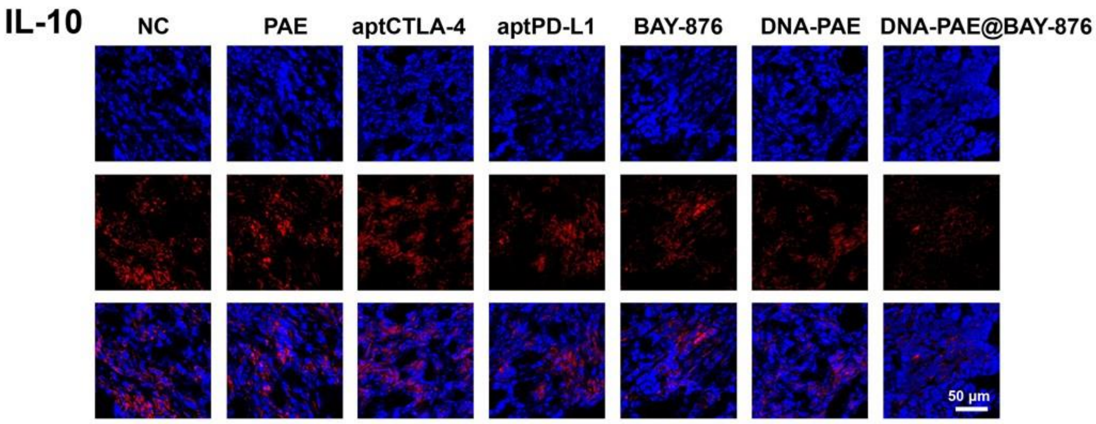

Supplementary Figure 38. IL-10 secretion levels in 4T1 tumors after 21 days of different treatments with five mice per group. Immunofluorescence experiments were repeated three times independently with similar results.

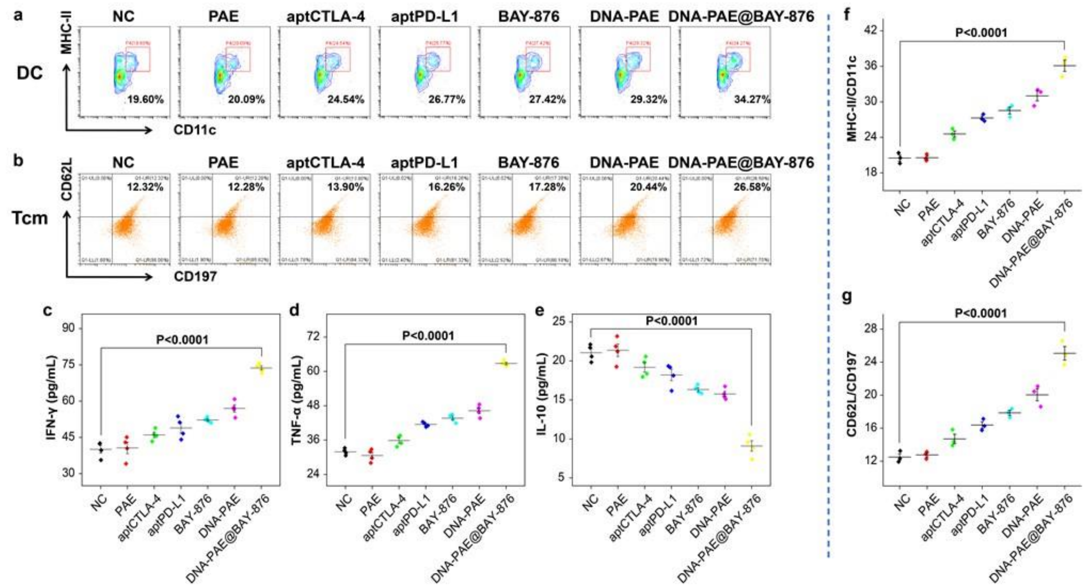

Supplementary Figure 39. Effector immune cell deployment (EICD) in 4T1 tumor-bearing mice after various treatment. (a) Flow cytometry analysis on the maturation status of lymph node-resident DCs (CD11c+MHC-II+). (b) Flow cytometry analysis on the lymph node-resident central memory T cell populations (CD197+CD62L+). (c-e) Serum cytokine levels in mice after 21 days of different treatment. (f-g) Statistical analysis of the flow cytometric data in panels a-b. Flow cytometry experiments in panels a and b were repeated three times independently with similar results. Data are presented as mean values ± SEM (n = 4 mice for panels c-e, n = 3 mice for panels f-g). Statistical analysis was carried out via two-way ANOVA method. Source data are provided as a Source Data file.

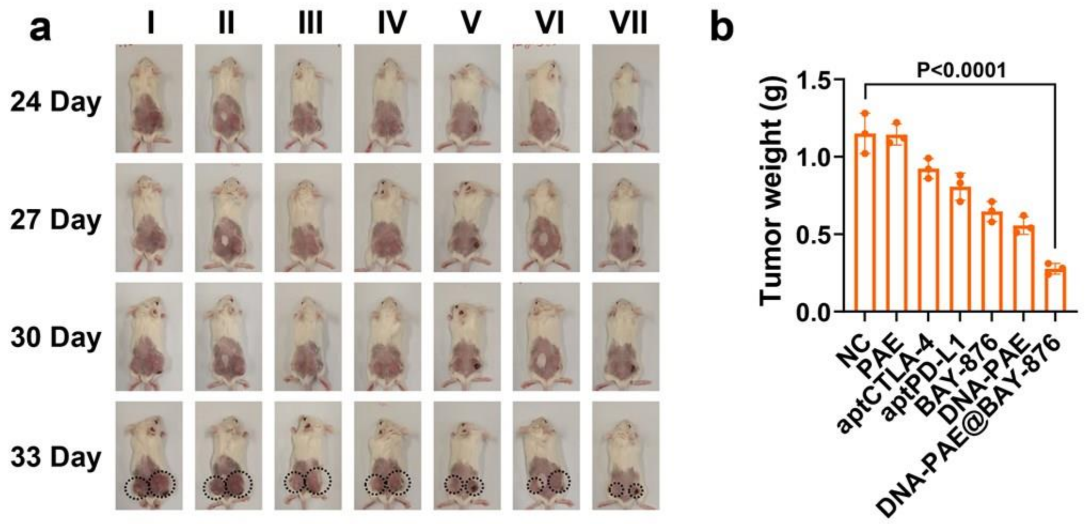

Supplementary Figure 40. Tumor changes on bilateral tumor models after treatment. (a) Photographical images of the bilateral tumors through the treatment period. (I) NC, (II) PAE, (III) aptCTLA-4, (IV) aptPD-L1, (V) BAY-876, (VI) DNA-PAE, (VII) DNA-PAE@BAY-876. (b) Weight comparison of the distal tumors from Balb/c mice after different treatment. Data are presented as mean values ± SEM (n = 3 mice for panels a-b). Statistical analysis was carried out via two-way ANOVA method. Source data are provided as a Source Data file.

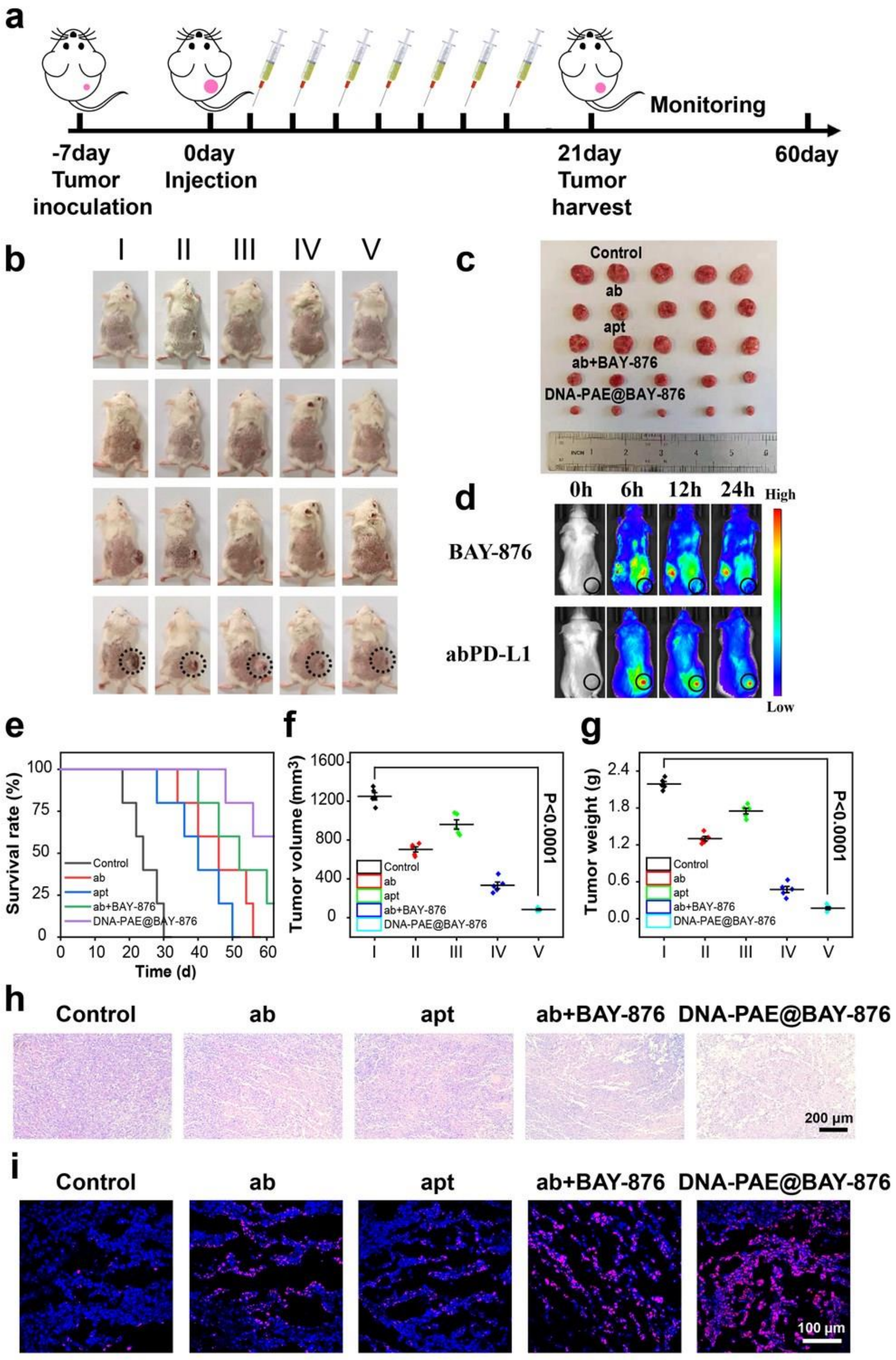

Supplementary Figure 41. Comparison of DNA-PAE@BAY-876 to anti-PD-L1/anti-CTLA-4 antibody combination in 4T1 tumor-bearing mice. (a) Schematic diagram of

the treatment schedule in vivo. (b) Photographical images of the 4T1 tumors through the treatment period. (I) Control, (II) ab, (III) apt, (IV) ab+BAY-876, (V) DNA-PAE@BAY-876. (c) Visual comparison of tumors from Balb/c mice after different treatment. (d) Systemic distribution of BAY-876/abPD-L1 at 0/6/12/24h post intravenous injection with three mice per group. (e) Survival curves of 4T1 tumor-bearing mice after different treatment. (f-g) Final tumor volume and weight in different groups at the end of the 21 days period. (h-i) H&E and TUNEL staining of tumor samples with five mice per group. ab was anti-PD-L1 antibody and anti-CTLA-4 antibody; apt was aptPD-L1 and aptCTLA-4. Experiments in panels b, d and h-i were repeated three times independently with similar results. Data are presented as mean values ± SEM (n = 5 mice for panels e-g). Statistical analysis was carried out via two-way ANOVA method. Source data are provided as a Source Data file.

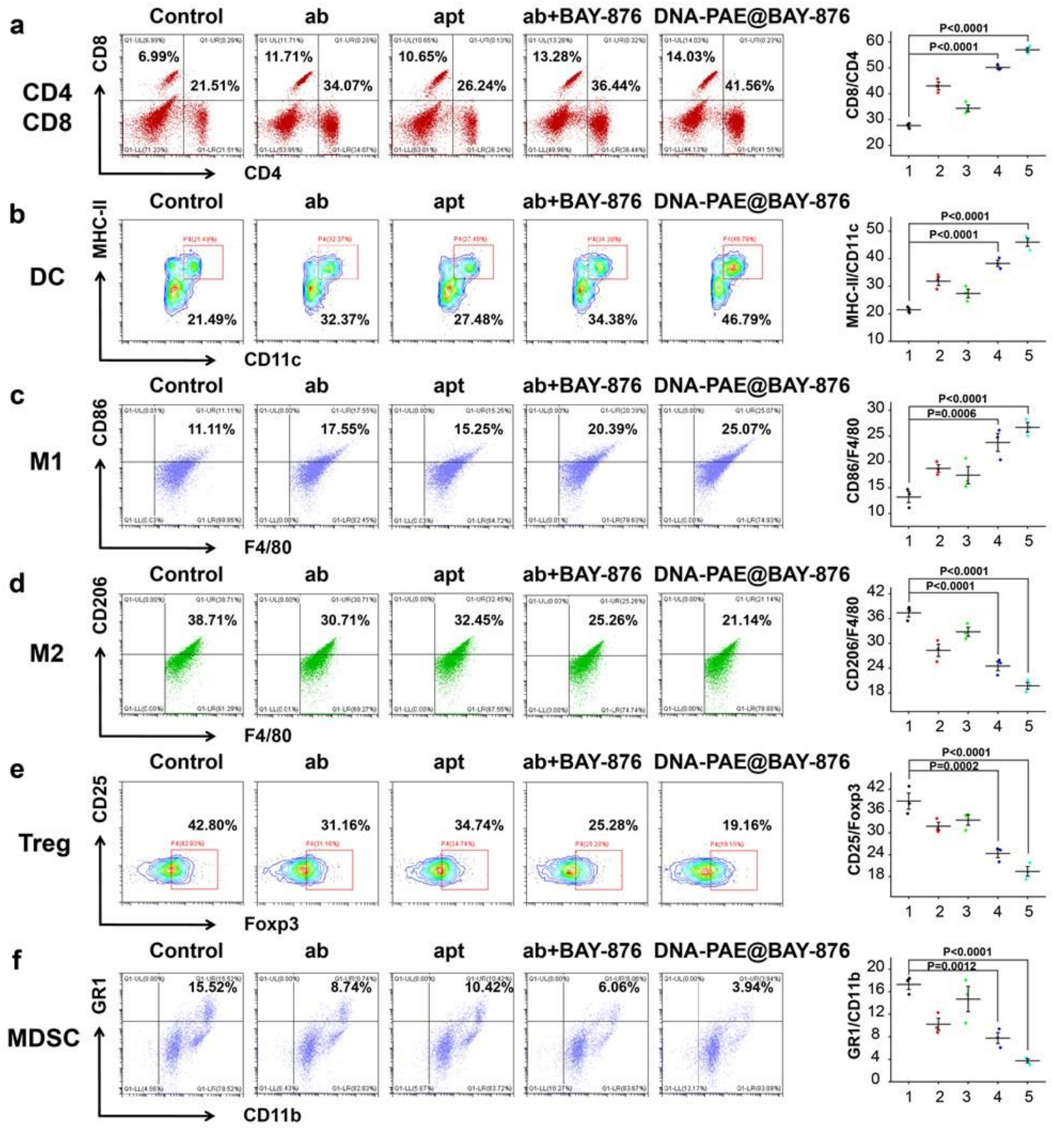

Supplementary Figure 42. Comparative analysis on the antitumor efficacy of DNA-PAE@BAY-876 and anti-PD-L1/anti-CTLA-4 antibody in 4T1 tumor-bearing mice. (a) Flow cytometry detection on the tumor infiltration of CD8+ T cells and CD4+ T cells. (b) Flow cytometry detection on the tumor infiltration of mature DCs (CD11c+MHC-II+). (c) Flow cytometry detection on the tumor-infiltration of M1 macrophages (F4/80+CD86+). (d) Flow cytometry detection on the tumor infiltration of M2 macrophages (F4/80+CD206+). (e) Flow cytometry detection on the tumor infiltration of Tregs (CD4+CD25+Foxp3+). (f) Flow cytometry detection on the tumor infiltration

of MDSCs (CD11b+GR1+). (1) Control, (2) ab, (3) apt, (4) ab+BAY-876, (5) DNA-PAE@BAY-876. ab was anti-PD-L1 antibody and anti-CTLA-4 antibody; apt was aptPD-L1 and aptCTLA-4. Flow cytometry experiments in panels a-f were repeated three times independently with similar results. Data are presented as mean values ± SEM (n = 3 mice for panels a-f). Statistical analysis was carried out via two-way ANOVA method. Source data are provided as a Source Data file.

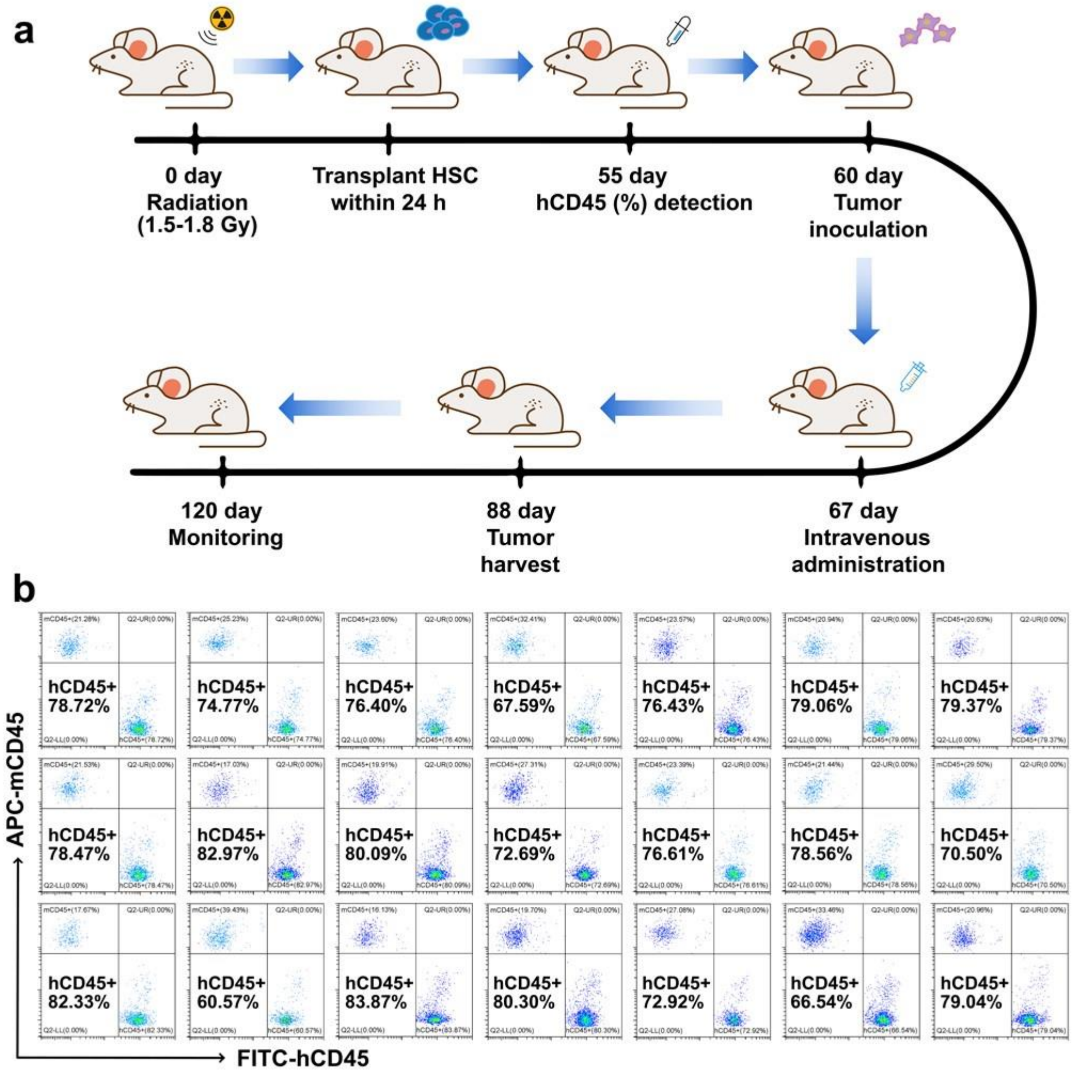

Supplementary Figure 43. Construction of humanized MDA-MB-231 tumor-bearing HSC-NOG-EXL mice. (a) Schematic diagram of humanized MDA-MB-231 tumor-bearing mice and the treatment schedule. (b) Flow cytometry validation of HSC-NOG-EXL mice by examining the peripheral blood after injection with HSC cells through the tail vein after 55 days, each panel represents the analysis result for one individual mouse. Flow cytometry experiments in panel b were repeated three times independently with similar results.

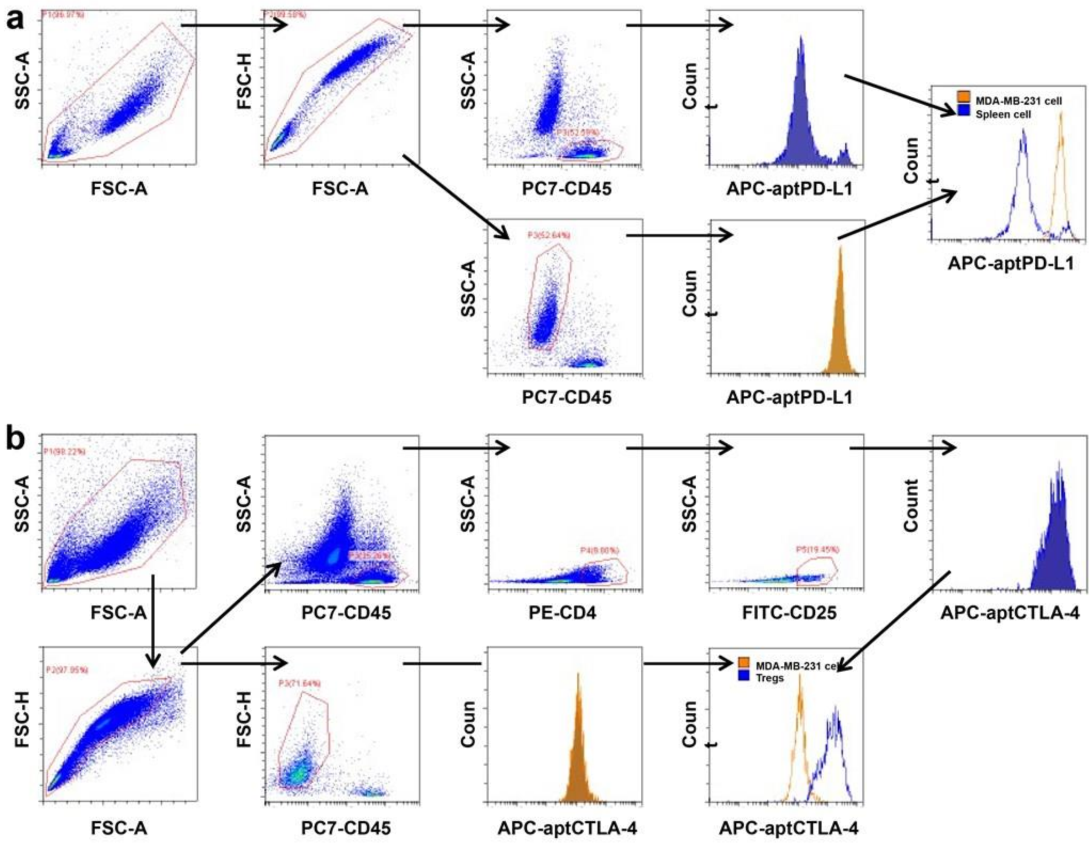

Supplementary Figure 44. Targeting ability of aptPD-L1 (a) and aptCTLA-4 (b) in the co-incubation system of MDA-MB-231 cells and splenic immune cells from humanized HSC-NOG-EXL mice. Flow cytometry experiments in panels a-b were repeated three times independently with similar results.

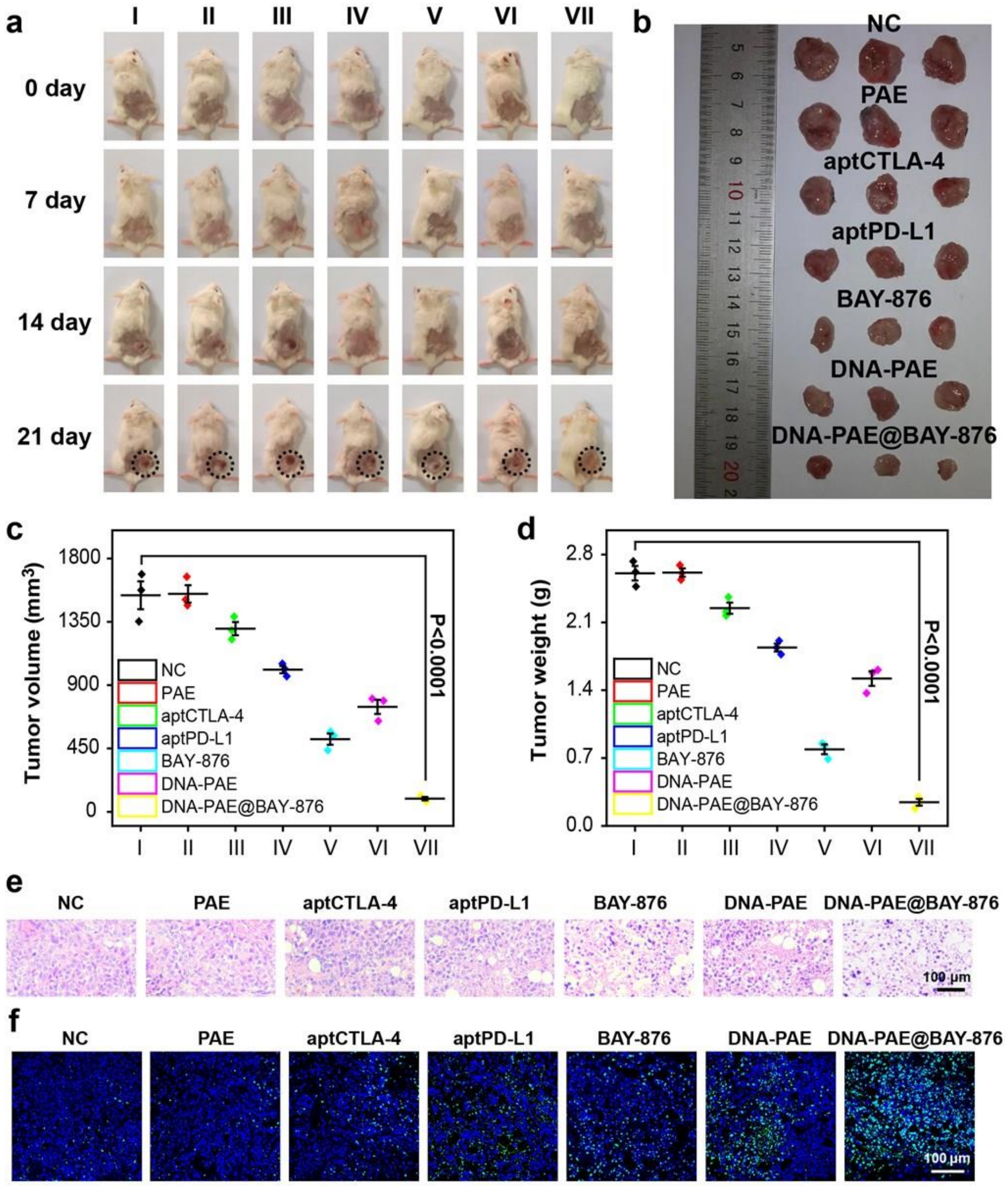

Supplementary Figure 45. Antitumor evaluation of DNA-PAE@BAY-876 in humanized MDA-MB-231 tumor-bearing HSC-NOG-EXL mice. (a) Photographical images of the MDA-MB-231 tumors through the treatment period. (b) Visual comparison of tumors from HSC-NOG-EXL mice after different treatment. (c-d) Final tumor volume and weight in different groups at the end of the 21 days period. (e-f) H&E and TUNEL staining of tumor samples with three mice per group. (I) NC, (II)

PAE, (III) aptCTLA-4, (IV) aptPD-L1, (V) BAY-876, (VI) DNA-PAE, (VII) DNA-PAE@BAY-876. Experiments in panels a and e-f were repeated three times independently with similar results. Data are presented as mean values ± SEM (n = 3 mice for panels c-d). Statistical analysis was carried out via two-way ANOVA method. Source data are provided as a Source Data file.

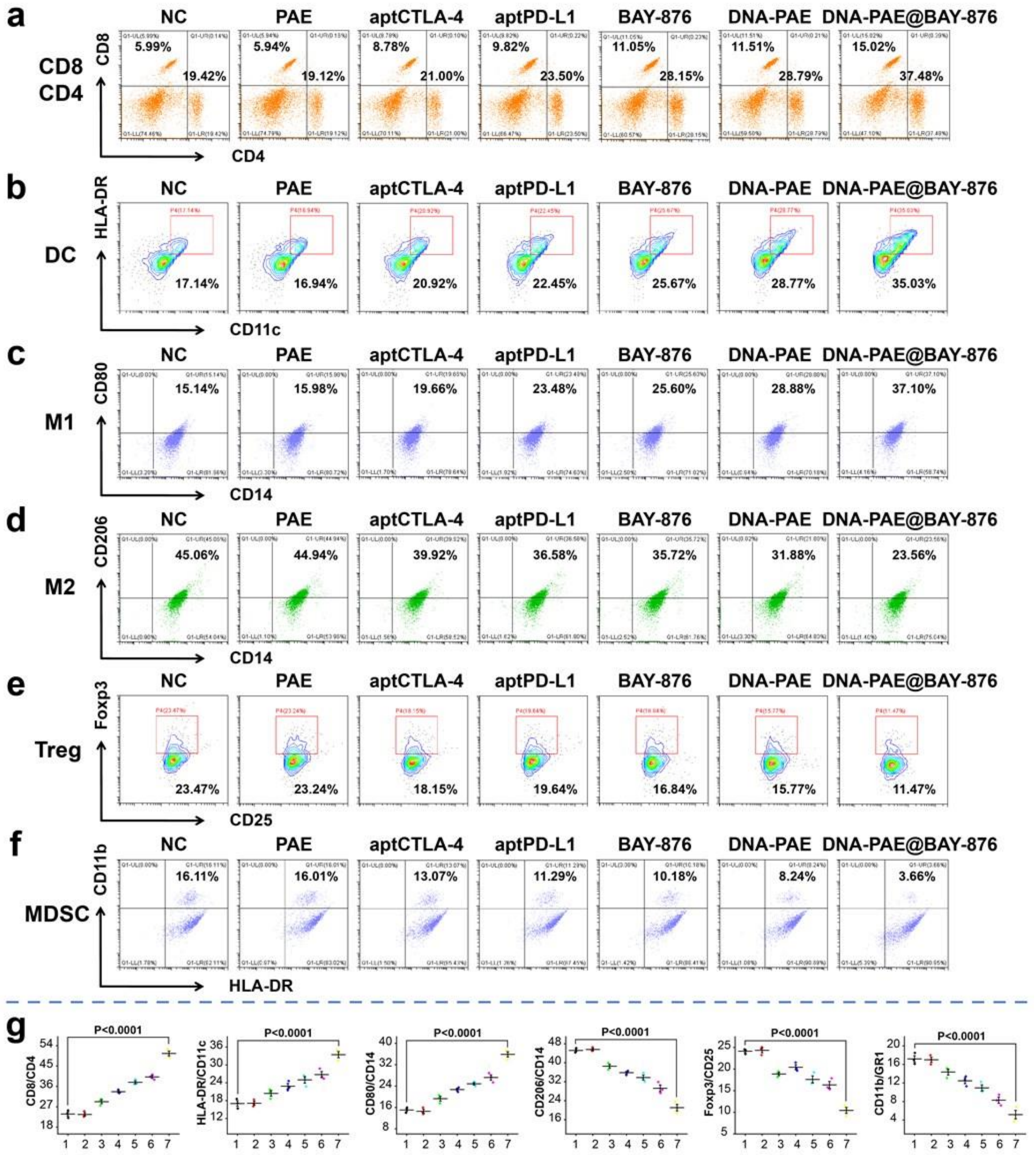

Supplementary Figure 46. Evaluation on DNA-PAE@BAY-876-mediated immunotherapeutic effect in MDA-MB-231 tumor-bearing humanized HSC-NOG-EXL mice. (a) Flow cytometry detection on the tumor infiltration of CD8+ T cells and CD4+ T cells. (b) Flow cytometry detection on the tumor infiltration of DCs (CD11c+HLA-DR+). (c) Flow cytometry detection on the tumor-infiltration of M1 macrophages (CD14+CD80+). (d) Flow cytometry detection on the tumor infiltration of M2 macrophages (CD14+CD206+). (e) Flow cytometry detection on the tumor

infiltration of Tregs (CD4+CD25+Foxp3+). (f) Flow cytometry detection on the tumor infiltration of MDSCs (HLA-DR-CD11b+). (g) Statistical analysis of the flow cytometric data in panels a-f. (1) NC, (2) PAE, (3) aptCTLA-4, (4) aptPD-L1, (5) BAY-876, (6) DNA-PAE, (7) DNA-PAE@BAY-876. Flow cytometry experiments in panels a-f were repeated three times independently with similar results. Data are presented as mean values ± SEM (n = 3 mice for panel g). Statistical analysis was carried out via two-way ANOVA method. Source data are provided as a Source Data file.

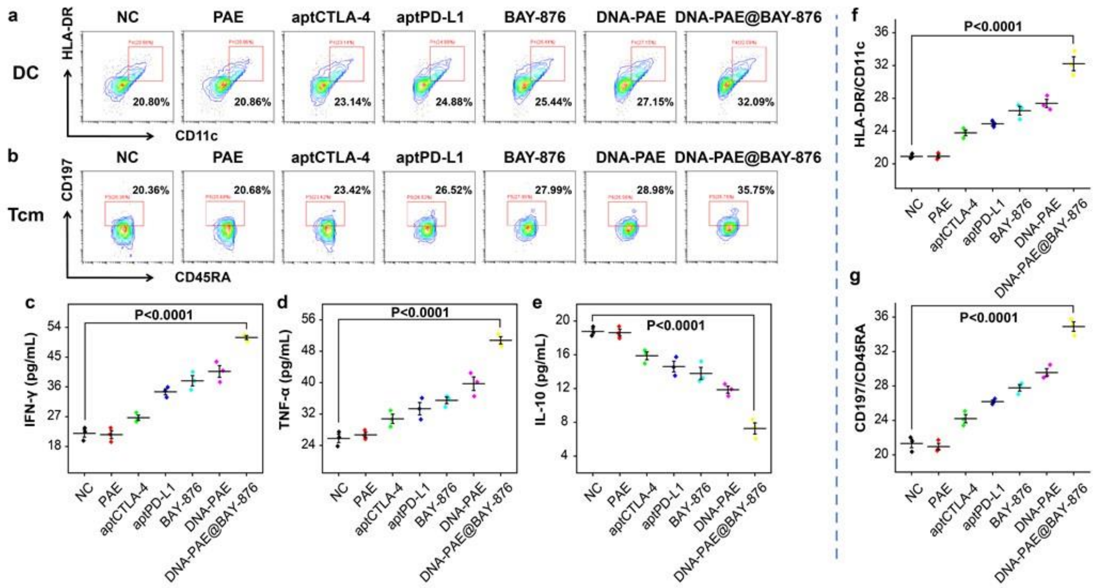

Supplementary Figure 47. Effector immune cell deployment (EICD) in MDA-MB-231 tumor-bearing humanized HSC-NOG-EXL mice. (a) Flow cytometry detection on the lymph node-resident DCs (CD11c+HLA-DR+). (b) Flow cytometry detection on the lymph node-resident central memory T cells (CD45RA-CD197+). (c-e) Cytokine secretion levels in humanized mice serum after 21 days of different treatment. (f-g) Statistical analysis of the flow cytometric data in panels a-b. Flow cytometry experiments in panels a-b were repeated three times independently with similar results. Data are presented as mean values ± SEM (n = 3 mice for panels c-g). Statistical analysis was carried out via two-way ANOVA method. Source data are provided as a Source Data file.

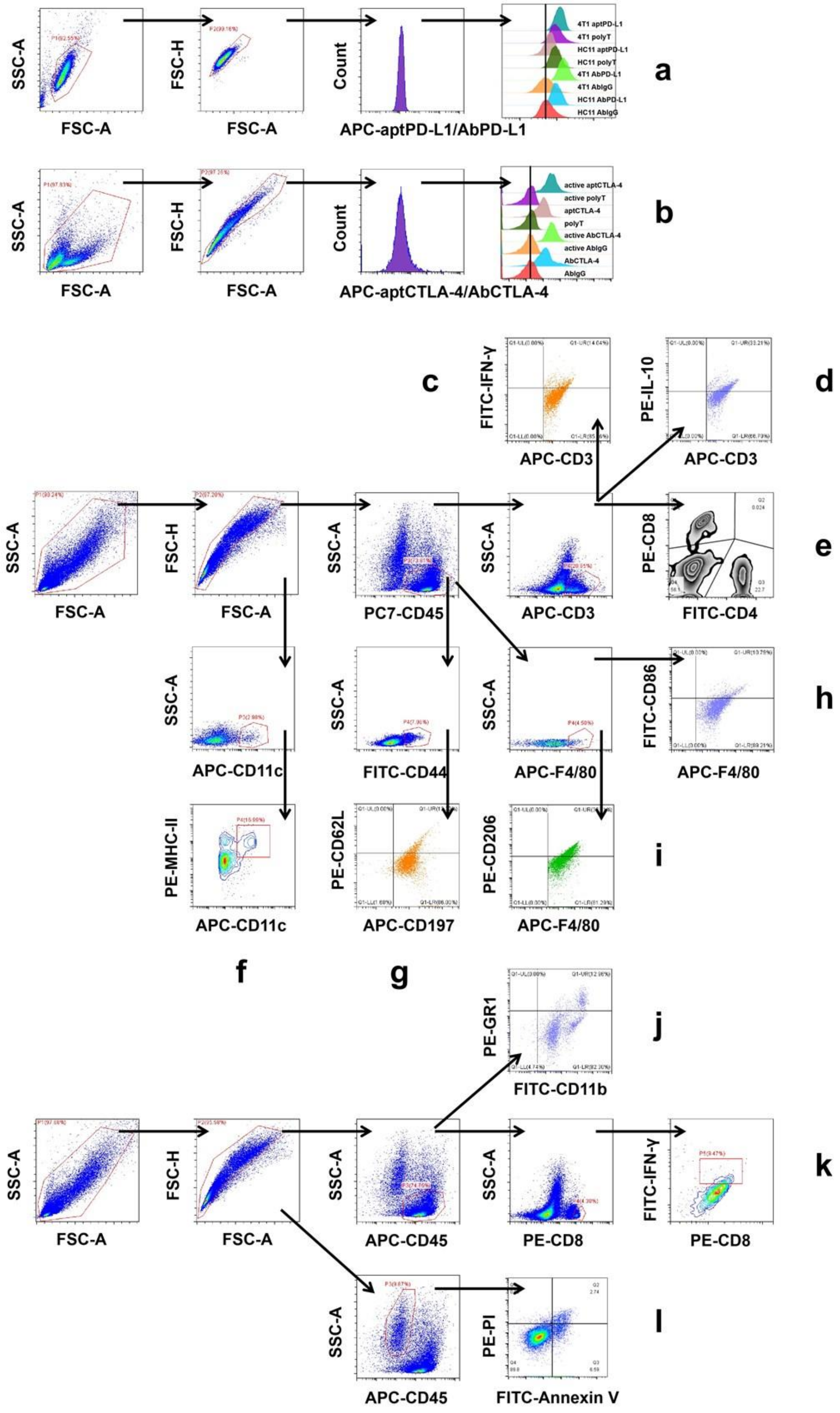
SSC-A
FSC-A
FSC-H
Count
APC-aptPD-L1/AbPD-L1
4T1 aptPD-L1
4T1 polyT
HC11 aptPD-L1
HC11 polyT
4T1 AbPD-L1
4T1 AbIgG
HC11 AbPD-L1
HC11 AbIgG
a
APC-aptCTLA-4/AbCTLA-4
active aptCTLA-4
active polyT
aptCTLA-4
polyT
active AbCTLA-4
active AbIgG
AbCTLA-4
AbIgG
b
FITC-IFN-γ
APC-CD3
c
PE-IL-10
d
PC7-CD45
PE-CD8
FITC-CD4
e
APC-CD11c
PE-MHC-II
f
FITC-CD44
PE-CD62L
APC-CD197
g
APC-F4/80
FITC-CD86
h
PE-CD206
i
PE-GR1
FITC-CD11b
j
APC-CD45
FITC-IFN-γ
PE-CD8
k
PE-PI
FITC-Annexin V
l

Supplementary Figure 48. Gating strategy for the FACS tests. a-b were applied for Figure 3a-b; c-d were applied for Supplementary Figure 29; e was applied for Figure 4f, Supplementary Figure 18a and Supplementary Figure 42a; f was applied for Supplementary Figure 18b, Supplementary Figure 21a, Supplementary Figure 35a, Supplementary Figure 39a and Supplementary Figure 42b; g was applied for Supplementary Figure 39b; h was applied for Figure 9f, Supplementary Figure 18c, Supplementary Figure 21b, Supplementary Figure 35b and Supplementary Figure 42c; i was applied for Supplementary Figure 35c and Supplementary Figure 42d; j was applied for Supplementary Figure 35d and Supplementary Figure 42f; k was applied for Figure 4g, Figure 8d, Figure 9e and Supplementary Figure 28b-c; l was applied for Figure 4a and Supplementary Figure 17.

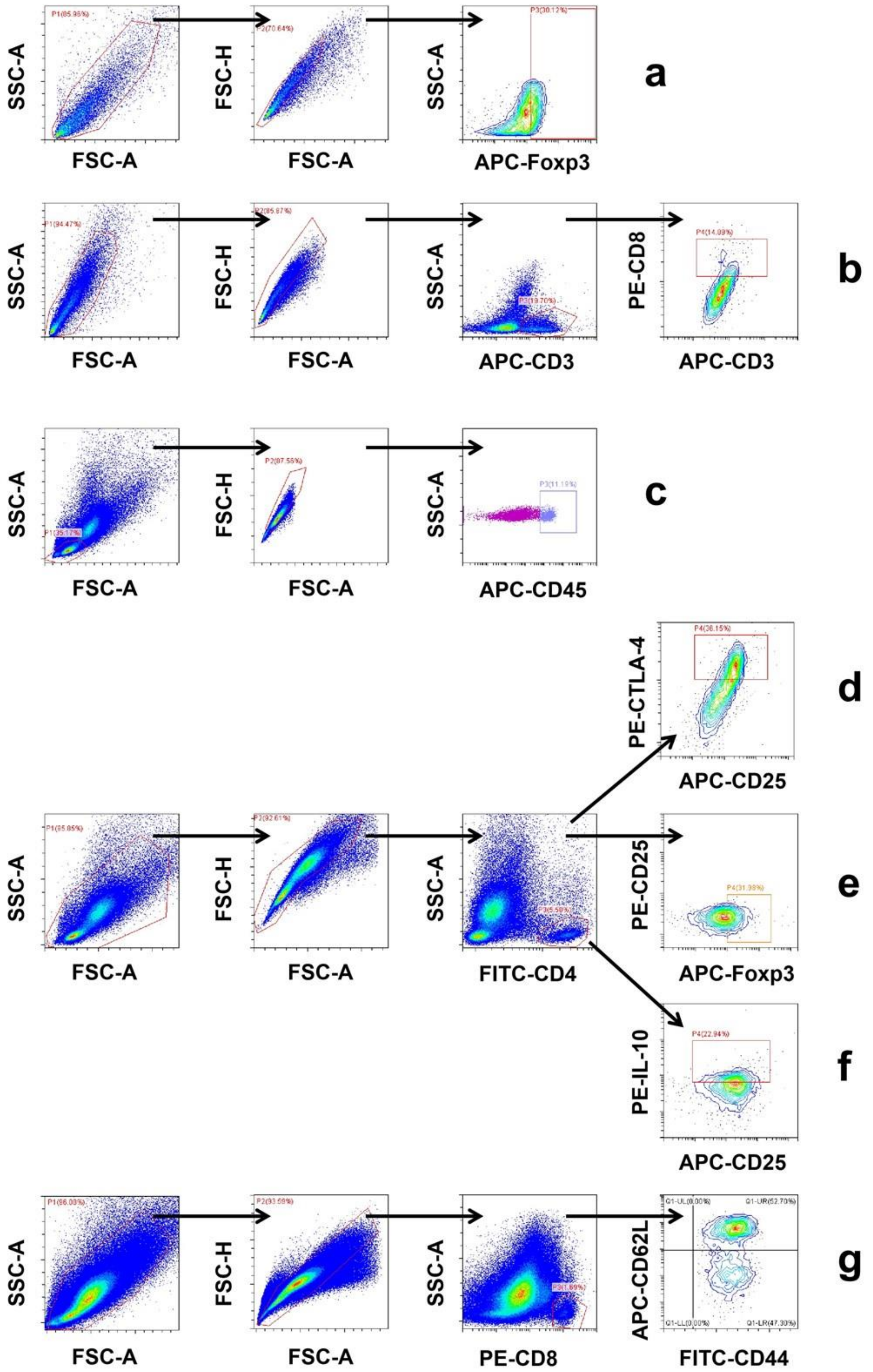

Supplementary Figure 49. Gating strategy for the FACS tests. Panel a was applied for Figure 6b; panel b was applied for Supplementary Figure 28a; panel c was applied for

Figure 8c and Figure 9d; panel d was applied for Figure 6c; panel e was applied for Figure 8a and Supplementary Figure 42; panel f was applied for Figure 8b; panel g was applied for Figure 9g.

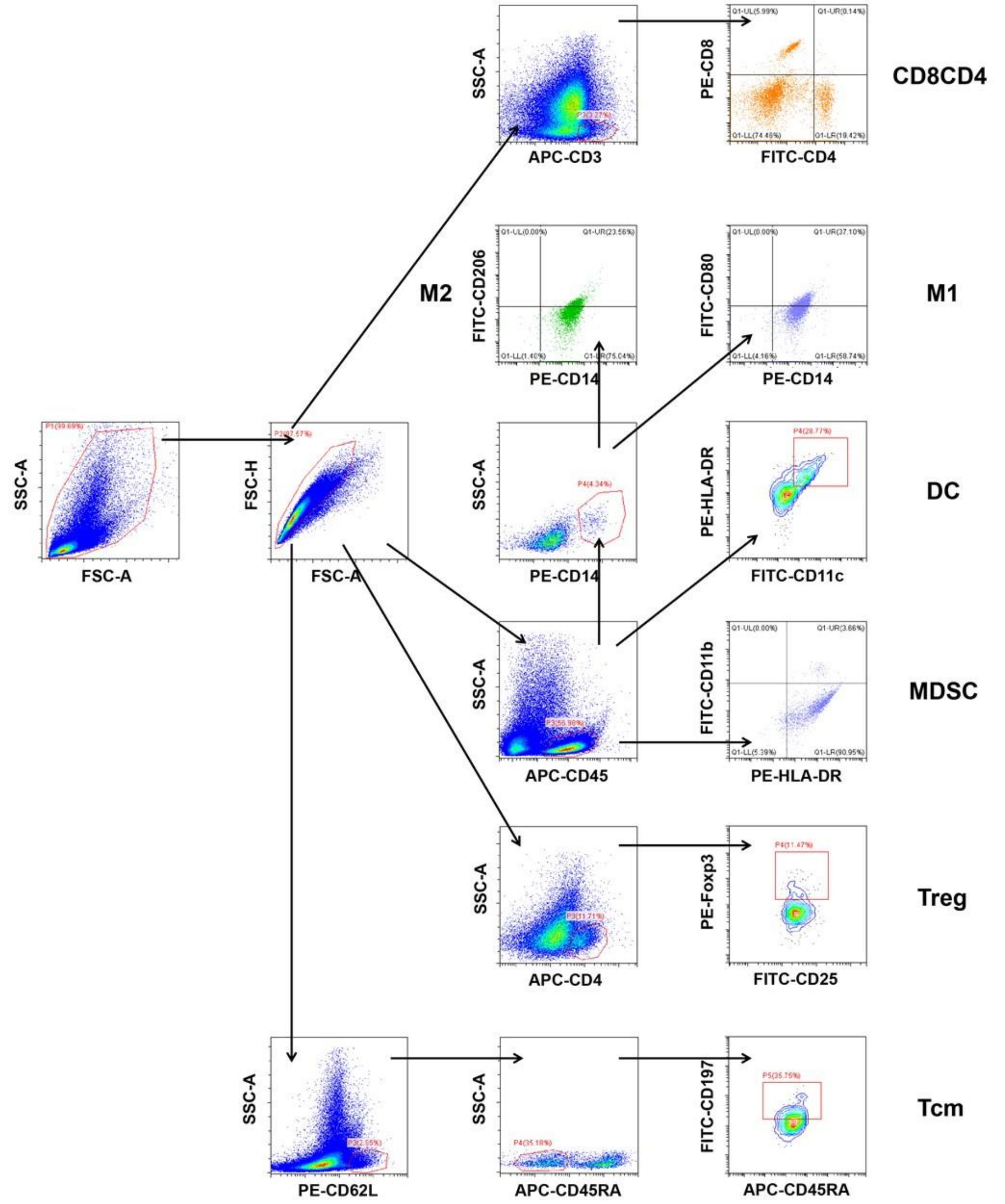

Supplementary Figure 50. Gating strategy for the FACS tests applied for Supplementary Figure 46 and Supplementary Figure 47.

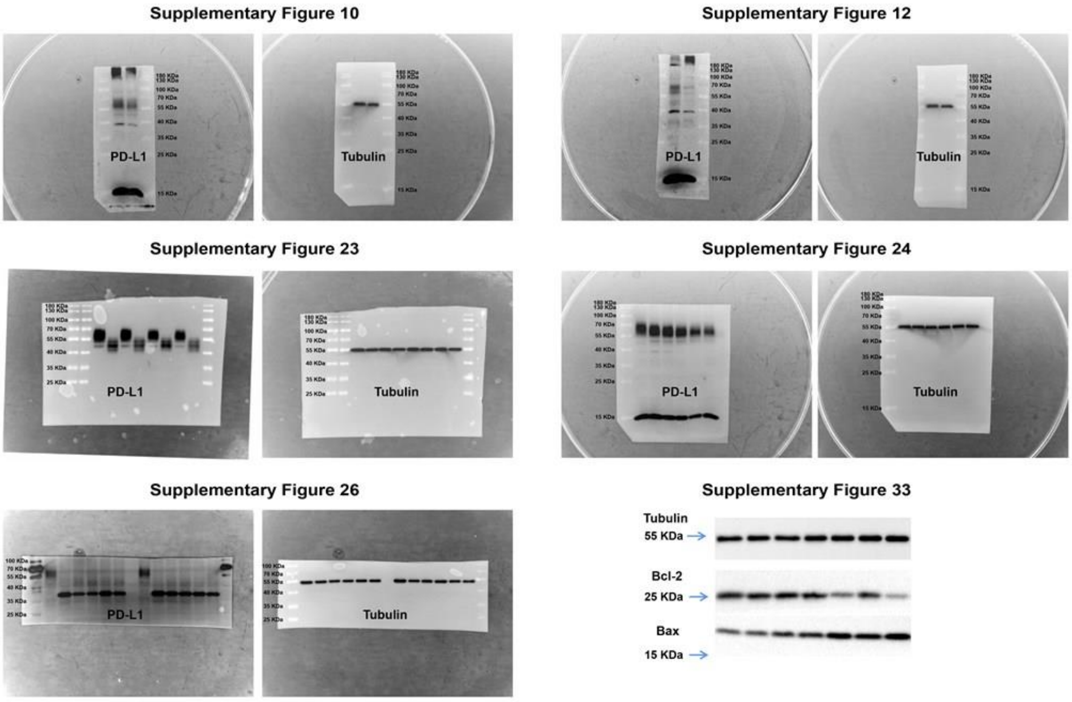

Supplementary Figure 51. Uncropped scans of western blot with marked molecular weight distributions for related supplementary figures.
