# Supplementary material for: Inhibition of glycolysis-driven immunosuppression with a nano-assembly enhances response to immune checkpoint blockade therapy in triple negative breast cancer
Source: Nat Commun. 2023 Nov 2;14:7021. doi: 10.1038/s41467-023-42883-2 (PMC10622423; doi:10.1038/s41467-023-42883-2)
Supplement: Supplementary file 3 — Reporting Summary [file 41467_2023_42883_MOESM3_ESM.pdf]

nature portfolio

Corresponding author(s): Menghuan Li, Zhong Luo, Yan Hu

Last updated by author(s): Apr 28, 2023

Reporting Summary

Nature Portfolio wishes to improve the reproducibility of the work that we publish. This form provides structure for consistency and transparency in reporting. For further information on Nature Portfolio policies, see our Editorial Policies and the Editorial Policy Checklist.

Statistics

For all statistical analyses, confirm that the following items are present in the figure legend, table legend, main text, or Methods section.

n/a	Confirmed	
☐	☒	The exact sample size (n) for each experimental group/condition, given as a discrete number and unit of measurement
☐	☒	A statement on whether measurements were taken from distinct samples or whether the same sample was measured repeatedly
☐	☒	The statistical test(s) used AND whether they are one- or two-sided<br>*Only common tests should be described solely by name; describe more complex techniques in the Methods section.*
☐	☒	A description of all covariates tested
☐	☒	A description of any assumptions or corrections, such as tests of normality and adjustment for multiple comparisons
☐	☒	A full description of the statistical parameters including central tendency (e.g. means) or other basic estimates (e.g. regression coefficient) AND variation (e.g. standard deviation) or associated estimates of uncertainty (e.g. confidence intervals)
☐	☒	For null hypothesis testing, the test statistic (e.g. F, t, r) with confidence intervals, effect sizes, degrees of freedom and P value noted<br>*Give P values as exact values whenever suitable.*
☒	☐	For Bayesian analysis, information on the choice of priors and Markov chain Monte Carlo settings
☒	☐	For hierarchical and complex designs, identification of the appropriate level for tests and full reporting of outcomes
☐	☒	Estimates of effect sizes (e.g. Cohen's d, Pearson's r), indicating how they were calculated

Our web collection on statistics for biologists contains articles on many of the points above.

Software and code

Policy information about availability of computer code

Data collection	LAS AF Lite, CytExpert
Data analysis	All statistical data were processed in GraphPad Prism (version 9.5 for Windows) by Student's t-test, one-way ANOVA or two-way ANOVA. All data was managed using Origin 2018 64Bit or GraphPad Prism (version 9.5 for Windows) . All figures are created by Adobe Illustrator 2020 and PowerPoint 2020.

For manuscripts utilizing custom algorithms or software that are central to the research but not yet described in published literature, software must be made available to editors and reviewers. We strongly encourage code deposition in a community repository (e.g. GitHub). See the Nature Portfolio guidelines for submitting code & software for further information.

Data

Policy information about availability of data

All manuscripts must include a data availability statement. This statement should provide the following information, where applicable:

- Accession codes, unique identifiers, or web links for publicly available datasets
- A description of any restrictions on data availability
- For clinical datasets or third party data, please ensure that the statement adheres to our policy

The authors declare that all data generated in this study are available within the Article, Supplementary Information or Source data file. Source data are provided with this paper.

Research involving human participants, their data, or biological material

Policy information about studies with human participants or human data. See also policy information about sex, gender (identity/presentation), and sexual orientation and race, ethnicity and racism.

Reporting on sex and gender	n/a
Reporting on race, ethnicity, or other socially relevant groupings	n/a
Population characteristics	n/a
Recruitment	n/a
Ethics oversight	n/a

Note that full information on the approval of the study protocol must also be provided in the manuscript.

Field-specific reporting

Please select the one below that is the best fit for your research. If you are not sure, read the appropriate sections before making your selection.

- [x] Life sciences
- [ ] Behavioural & social sciences
- [ ] Ecological, evolutionary & environmental sciences

For a reference copy of the document with all sections, see nature.com/documents/nr-reporting-summary-flat.pdf

Life sciences study design

All studies must disclose on these points even when the disclosure is negative.

Sample size	Sample size was determined based on the set-ups in previous reports to ensure adequate power (Nat Commun. 2022, 13(1), 5685).
Data exclusions	No data exclusion was performed.
Replication	All measurements were performed on three or more independent replicates from separate experiments.
Randomization	Samples and cells were randomly allocatad into groups. Mice with comparable age, weight and tumor sizes were randomly selected from the housing cages and then divided into experimental groups with no bias for further treatment.
Blinding	Investigators were blinded to group allocation during all experiments.

Reporting for specific materials, systems and methods

We require information from authors about some types of materials, experimental systems and methods used in many studies. Here, indicate whether each material, system or method listed is relevant to your study. If you are not sure if a list item applies to your research, read the appropriate section before selecting a response.

Materials & experimental systems

n/a	Involved in the study	
[ ]	[x]	Antibodies
[ ]	[x]	Eukaryotic cell lines
[x]	[ ]	Palaeontology and archaeology
[ ]	[x]	Animals and other organisms
[x]	[ ]	Clinical data
[x]	[ ]	Dual use research of concern
[x]	[ ]	Plants

Methods

n/a	Involved in the study	
[x]	[ ]	ChIP-seq
[ ]	[x]	Flow cytometry
[x]	[ ]	MRI-based neuroimaging

Antibodies

Antibodies used	PC7-anti-mouse CD45 antibody, APC-anti-mouse CD45 antibody, APC-anti-mouse CD3 antibody, APC-anti-mouse CD11c antibody, PE-anti-mouse CD8a antibody, FITC-anti-mouse CD4 antibody, APC-anti-mouse F4/80-antibody, PE-anti-mouse CD206 antibody, FITC-anti-mouse CD86 antibody, FITC-anti-mouse IFN-γ antibody, PE-anti-mouse IL-10 antibody, FITC-anti-mouse CD44 antibody, PE-anti-mouse MHC-II antibody, PE-anti-mouse CD62L antibody, PE-anti-mouse GR1 antibody, FITC-anti-mouse CD11b antibody, APC-anti-mouse CD25 antibody, PE-anti-mouse CD25 antibody, APC-anti-mouse CD62L antibody, FITC-anti-human CD206 antibody, PE-anti-human CD14 antibody, PE-anti-human HLA-DR antibody, FITC-anti-human CD11c antibody, APC-anti-human CD45 antibody, FITC-

anti-human CD11b antibody, APC-anti-human CD4 antibody, FITC-anti-human CD25 antibody, PE-anti-human CD62L antibody, APC-anti-human CD45RA antibody, FITC-anti-human CD197 antibody, FITC-anti-human CD4 antibody, PE-anti-human CD8a antibody, APC-anti-human CD3 antibody were purchased from elabscience (Wuhan, China), of which the catalog numbers were E-AB-F1136G, E-AB-F1136E, E-AB-F1013E, F-AB-F0991E, E-AB-F1104D, E-AB-F1353C, E-AB-F0995E, E-AB-F1135D, E-AB-F0994C, E-AB-F1101C, E-AB-F1197D, E-AB-F1100C, E-AB-F0990D, E-AB-F1011D, EAB-F1120D, E-AB-F1081C, E-AB-F1102E, E-AB-F1102D, E-AB-F1011E, E-AB-F1161C, E-AB-F1209D, E-AB-F1111D, E-AB-F1118C, E-AB-F1137E, E-AB-F1146C, E-AB-F1109E, E-AB-F1194C, E-AB-F1051D, E-AB-F1052E, E-AB-F1159C, E-AB-F1352C, E-AB-F1110D, E-AB-F1001E, respectively. The dilution ratios of above-mentioned antibodies were 1:50.

PE-anti-mouse CTLA-4 antibody, anti-mouse PD-L1 antibody, APC-anti-mouse PD-L1 antibody, FITC-anti-CTLA-4 antibody were purchased from Abcam (Shanghai, China), of which the catalog numbers were ab210383 (1:100), ab213480 (1:1000), ab272329 (1:100), ab24935 (1:100), respectively.

anti-mouse Bcl-2 antibody and anti-mouse Bax antibody were purchased from Zenbio (Chengdu, China), of which the catalog numbers were 381702 (1:1000), R23596 (1:1000), respectively.

anti-mouse β-Tubulin antibody was purchased from Abmart (Shanghai, China), of which the catalog number was M30109S (1:1000), respectively.

APC-anti-mouse CD197 antibody, APC-anti-mouse CTLA-4 antibody, FITC-anti-human CD80 antibody and PE-anti-human Foxp3 antibody were purchased from Biolegend (Beijing, China), of which the catalog numbers was 120107 (1:50), 106309 (1:50), 375405 (1:50) and 364703 (1:50), respectively.

APC-anti-mouse Foxp3 antibody was purchased from proteintech (Wuhan, China), of which the catalog number was APC-65089 (1:50).

InVivoMAb anti-human PD-L1 (B7-H1) and InVivoMAb anti-human CTLA-4 (CD152) were purchased from bioxcell (Suzhou, China), of which the catalog numbers was BE0285 (50 μg·mL-1·g-1) and BE0190 (50 μg·mL-1·g-1), respectively.

Validation

All antibodies in the study were freshly obtained from the manufacturers and used according to the user manuals.

PC7-anti-CD45 antibody https://www.elabscience.cn/p-pe_cyanine5_anti_mouse_cd45_antibody_30_f11_-172697.html
APC-anti-CD45 antibody https://www.elabscience.cn/p-apc_anti_mouse_cd45_antibody_30_f11_-172695.html
APC-anti-CD3 antibody https://www.elabscience.cn/p-apc_anti_mouse_cd3_antibody_17a2_-133030.html
APC-anti-CD11c antibody https://www.elabscience.cn/p-apc_anti_mouse_cd11c_antibody_n418_-132974.html
PE-anti-CD8a antibody https://www.elabscience.cn/p-pe_anti_mouse_cd8a_antibody_53_6.7_-133053.html
FITC-anti-CD4 antibody https://www.elabscience.cn/p-fitc_anti_mouse_cd4_antibody_rm4_5_-531529.html
APC-anti-F4/80 antibody https://www.elabscience.cn/p-apc_anti_mouse_f4_80_antibody_ci_a3_1_-132982.html
PE-anti-CD206 antibody https://www.elabscience.cn/p-pe_anti_mouse_cd206_antibody_c068c2_-147870.html
FITC-anti-CD86 antibody https://www.elabscience.cn/p-fitc_anti_mouse_cd86_antibody_gl_1_-149393.html
FITC-anti-IFN-γ antibody https://www.elabscience.cn/p-fitc_anti_mouse_ifn_gamma_antibody_xmg1.2_-172590.html
PE-anti-IL-10 antibody https://www.elabscience.cn/p-pe_anti_mouse_il_10_antibody_jes5_16e3_-172898.html
FITC-anti-CD44 antibody https://www.elabscience.cn/p-fitc_anti_human_mouse_cd44_antibody_im7_-176555.html
PE-anti-MHC-II antibody https://www.elabscience.cn/p-pe_anti_mouse_mhc_ii_i_a_i_e_antibody_m5_114_-132965.html
PE-anti-CD62L antibody https://www.elabscience.cn/p-pe_anti_mouse_cd62l_antibody_mel14_-134631.html
PE-anti-GR1 antibody https://www.elabscience.cn/p-pe_anti_mouse_ly_6g_ly_6c_gr_1_antibody_rb6_8c5_-150590.html
FITC-anti-CD11b antibody https://www.elabscience.cn/p-fitc_anti_mouse_human_cd11b_antibody_m1_70_-133068.html
APC-anti-Foxp3 antibody https://www.ptgcn.com/products/Foxp3-Antibody-APC-65089.htm
PE-anti-CTLA-4 antibody https://www.abcam.cn/products/primary-antibodies/pe-ctla4-antibody-uc10-4f10-11-ab210383.html
APC-anti-CD25 antibody https://www.elabscience.cn/p-apc_anti_mouse_cd25_antibody_pc_61.5.3_-134626.html
PE-anti-CD25 antibody https://www.elabscience.cn/p-pe_anti_mouse_cd25_antibody_pc_61.5.3_-172594.html
APC-anti-CD62L antibody https://www.elabscience.cn/p-apc_anti_mouse_cd62l_antibody_mel14_-172281.html
APC-anti-CD197 antibody https://www.biolegend.com/en-us/products/apc-anti-mouse-cd197-ccr7-antibody-2822
anti-PD-L1 antibody https://www.abcam.cn/products/primary-antibodies/pd-l1-antibody-epr20529-ab213480.html
APC-anti-PD-L1 antibody https://www.abcam.cn/products/primary-antibodies/apc-pd-l1-antibody-10f9g2-ab272329.html
FITC-anti-CTLA-4 antibody https://www.abcam.cn/products/primary-antibodies/fitc-ctla4-antibody-1b8-ab24935.html
APC-anti-CTLA-4 antibody https://www.biolegend.com/en-us/products/apc-anti-mouse-cd152-antibody-5455
anti-Bcl-2 antibody http://www.zen-bio.cn/prod_view.aspx?IsActiveTarget=True&TypeId=180&Id=539422&FId=t3:180:3
anti-Bax antibody http://www.zen-bio.cn/prod_view.aspx?IsActiveTarget=True&TypeId=171&Id=533394&FId=t3:171:3
anti-β-Tubulin antibody http://www.ab-mart.com.cn/page.aspx?node=%2059%20&id=%20992
FITC-anti-CD206 antibody https://www.elabscience.cn/p-fitc_anti_human_cd206_antibody_15_2_-172796.html
PE-anti-CD14 antibody https://www.elabscience.cn/p-pe_anti_human_cd14_antibody_m5e2_-172927.html
FITC-anti-CD80 antibody https://www.biolegend.com/en-us/products/fitc-anti-human-cd80-antibody-20587
PE-anti-HLA-DR antibody https://www.elabscience.cn/p-pe_anti_human_hla_dr_antibody_l243_-133109.html
FITC-anti-CD11c antibody https://www.elabscience.cn/p-fitc_anti_human_cd11c_antibody_bu15_-133020.html
APC-anti-CD45 antibody https://www.elabscience.cn/p-apc_anti_human_cd45_antibody_hi30_-172704.html
FITC-anti-CD11b antibody https://www.elabscience.cn/p-fitc_anti_human_cd11b_antibody_icrf44_-172748.html
APC-anti-CD4 antibody https://www.elabscience.cn/p-apc_anti_human_cd4_antibody_rpa_t4_-133094.html
PE-anti-Foxp3 antibody https://www.biolegend.com/en-us/products/pe-anti-human-foxp3-recombinant-antibody-20719
FITC-anti-CD25 antibody https://www.elabscience.cn/p-fitc_anti_human_cd25_antibody_bc96_-172888.html
PE-anti-CD62L antibody https://www.elabscience.cn/p-pe_anti_human_cd62l_antibody_dreg56_-172422.html
APC-anti-CD45RA antibody https://www.elabscience.cn/p-apc_anti_human_cd45ra_antibody_hi100_-134570.html
FITC-anti-CD197 antibody https://www.elabscience.cn/p-fitc_anti_human_cd197_ccr7_antibody_g043h7_-172791.html
FITC-anti-CD4 antibody https://www.elabscience.cn/p-fitc_anti_human_cd4_antibody_sk3_-531520.html
PE-anti-CD8a antibody https://www.elabscience.cn/p-pe_anti_human_cd8a_antibody_okt_8_-133101.html
APC-anti-CD3 antibody https://www.elabscience.cn/p-apc_anti_human_cd3_antibody_okt_3_-132990.html
InVivoMAb anti-human PD-L1 (B7-H1) https://bioxcell.com/invivomab-anti-human-pd-l1-b7-h1

InVivoMAb anti-human CTLA-4 (CD152) https://bioxcell.com/invivomab-anti-human-ctla-4-cd152-be0190

Eukaryotic cell lines

Policy information about cell lines and Sex and Gender in Research

Cell line source(s)	4T1, HC11, B16F10, 3T3, 4T1-luc, MDA-MB-231 cell lines were purchased from Shanghai Zeye Biotechnology Co., Ltd. with the catalog number of ZY-C6054M, ZY-C6027M, ZY-C6002M, ZY-C6050M, ZY-C6101M, ZY-C6044H, respectively.
Authentication	These cell lines were authenticated by the supplier using STR analysis.
Mycoplasma contamination	No contamination was detected by the supplier using Hoechst DNA stain method, agar culture method and PCR-based assay.
Commonly misidentified lines (See ICLAC register)	No commonly misidentified cell lines were used in the study.

Animals and other research organisms

Policy information about studies involving animals; ARRIVE guidelines recommended for reporting animal research, and Sex and Gender in Research

Laboratory animals	C57BL/6J and Balb/c mice (female, 6-week-old) were provided by Hunan Slake Jingda Experimental Animal Co., Ltd. The humanized HSC-NOG-EXL mice (female, 17-week-old) were provided by Beijing Charles River Experimental Animal Technology Co., Ltd.<br>All mice were kept in the animal house of Chongqing Medical University. Mice were housed in cages with five mice per cage and kept on in a regular 12 h light: 12 h dark cycle (9:00–21:00; 21:00–9:00). The temperature was 22 ± 1 degree Celsius and humidity was 40–68%.
Wild animals	No wild animals were used in the study.
Reporting on sex	Female mice were used in this study to eliminate potential experimental bias.
Field-collected samples	No field collected samples were used in the study.
Ethics oversight	All animal tests have been reviewed and approved by the Animal Care and Use Committee of Laboratory Animals Administration of Chongqing Medical University, which were carried out following the Animal Management Rules of the Ministry of Health of the People's Republic of China.

Note that full information on the approval of the study protocol must also be provided in the manuscript.

Flow Cytometry

Plots

Confirm that:

- [x] The axis labels state the marker and fluorochrome used (e.g. CD4-FITC).
- [x] The axis scales are clearly visible. Include numbers along axes only for bottom left plot of group (a 'group' is an analysis of identical markers).
- [x] All plots are contour plots with outliers or pseudocolor plots.
- [x] A numerical value for number of cells or percentage (with statistics) is provided.

Methodology

Sample preparation	Determination of dissociation constant of aptCTLA-4 or aptPD-L1: 1 mL streptavitin-coated magnetic beads were stood for 3 min with the magnet, then washed by PBS for three times. The biotin-modified mouse CTLA-4 or mouse PD-L1 protein with a final concentration of 50 nM was mixed with the above magnetic beads, and incubated on the rotator for 60 min. After cleaning with PBS for three times, FAM-aptCTLA-4 or Cy5-aptPD-L1 with different concentrations were added into the above mixture, then incubated at room temperature for 30 min. After washing twice with PBS, the fluorescence level was measured by flow cytometry (CytoFLEX, Beckman Coulter). The dissociation constants of aptPD-L1 and aptCTLA-4 were calculated by the formula Y = Bmax X / (KD + X).<br>aptPD-L1 and aptCTLA-4 binding: 4T1 cells/HC11 cells were inoculated into 12-well plate with 1.5×105 per well. After culturing for 24 h, the cells were blocked with 5% BSA for 30 min. Subsequently, different fluorescently labeled samples were added into above cells. After incubated for 30 min, these cells were detected by flow cytometry (CytoFLEX, Beckman Coulter).<br>T cells were sorted from splenocytes of C57BL/6J mice, and some of them were activated by antibody treatment. T cells were stored in 1.5 mL centrifuge tubes and then blocked with 5% BSA for 30 min. Different fluorescently labeled samples were added into above T cells and incubated for 30 min. Finally, these T cells were detected by flow cytometry (CytoFLEX, Beckman Coulter).<br>Binding of aptPD-L1 and aptCTLA-4 in co-culture system: 4T1 cells and splenic immune cells were mixed in the 1.5 mL centrifuge tube at a ratio of 1:30 and treated with 5% BSA for 30 min, followed by the treatment of Cy5-aptPD-L1 or FAM-

aptCTLA-4 for another 30 min of incubation. After washing twice with PBS, PC7-anti-CD45 antibody, PE-anti-CD4 antibody and APC-anti-CD25 antibody was added into above cells. Finally, the fluorescence intensity on 4T1 cells or immune cells was measured by flow cytometry (CytoFLEX, Beckman Coulter).

Quantitative analysis of immunofluorescence images for determining GLUT1 and GLUT3 expression: The 4T1 tumor-bearing Balb/c mouse model was constructed, and the tumors sized about 600 mm3 were collected. The 4T1 tumor was washed twice with PBS, placed on a 0.4 μm filter membrane with adding high-glucose DMEM medium, and carefully pulverized with the syringe head, then mixed with 5 mL red blood cell lysis buffer and stood for 10 min, and centrifuged at 2000 rpm for 5 min. The cells were collected and sealed by 10% FBS for 2 h, then mixed with anti-GLUT1 antibody (rabbit host) and anti-GLUT3 antibody (mouse host) overnight at 4°C, followed by incubation with Cy5-labeled rabbit second antibody or FAM-labeled mouse second antibody for 2 h at room temperature. After washing twice with PBS, the cells were assayed by flow cytometry (CytoFLEX, Beckman Coulter).

Glucose uptake analysis of 4T1 cells and T cells: 4T1 cells were inoculated in 12-well plate with 1.5×105 cells per well, cultured for 24 h, added with different samples and T cells of 30 times the amount of tumor cells. 30 mM 2-NBDG was subsequently added and incubated at 37°C for 24 h. After centrifuging at 1500 rpm for 5 min, all cells were added with APC-anti-CD45 antibody, incubated at room temperature for 30 min in the dark, then detected by flow cytometry (CytoFLEX, Beckman Coulter).

4T1 cells were inoculated in 12-well plate with 1.5×105 cells per well, then added with different concentrations of BAY-876 after cultured for 24 h. 30 mM 2-NBDG was subsequently added and incubated at 37°C for 24 h. Finally, 4T1 cells were collected and detected by flow cytometry (CytoFLEX, Beckman Coulter).

Evaluation of the impact of BAY-876 and KL-11743 on major immune cell populations in the co-incubation system: 4T1 cells were inoculated into 12-well plate with 1.5×105 units per well. The cells were cultured for 24 h, added with splenocytes of 30 times the amount of tumor cells, and then incubated with 100 nM BAY-876 or 5 μM KL-11743 at 37°C for 24 h. The splenocytes were collected and processed with the corresponding fluorescent antibodies at room temperature for 30 min in the dark. Finally, the cells were detected by flow cytometry (CytoFLEX, Beckman Coulter).

4T1 cell apoptosis assay: 4T1 cells were inoculated into 12-well plate with 1.5×105 per well. The cells were cultured for 24 h, added with different samples and splenocytes of 30 times the amount of tumor cells, and incubated at 37°C for 24 h. After centrifuging at 1500 rpm for 5 min, all cells were added with 5 μL FITC-Annexin V and APC-anti-CD45 antibody, incubated at room temperature for 30 min in the dark, then added with 10 μL PI and incubated at room temperature for 10 min in the dark. The stained cells were finally detected by flow cytometry (CytoFLEX, Beckman Coulter).

T cell apoptosis assay: T cells were separated from splenocytes and added into 12-well plate with equal amount, followed by the addition of different samples. The cells were incubated in an incubator for 24 h. The T cells were centrifuged at 2000 rpm for 5 min and added with 5 μL FITC-Annexin V and APC-anti-CD3 antibody. After incubation at room temperature for 30 min in the dark, the T cells were added with 10 μL PI and then incubated at room temperature for 10 min in the dark before the detection by flow cytometry (CytoFLEX, Beckman Coulter).

Evaluation of the phenotypical changes of major immune cell populations: 4T1 cells were inoculated into 12-well plate with 1.5×105 units per well. The cells were cultured for 24 h, added with different samples and splenocytes of 30 times the amount of tumor cells, and then incubated at 37°C for 24 h. The splenocytes were collected and processed by the corresponding fluorescent antibodies at room temperature for 30 min in the dark. Finally, the cells were detected by flow cytometry (CytoFLEX, Beckman Coulter).

PD-1/aptPD-L1 competition assay: 4T1 cells were inoculated into 12-well plate with 1.5×105 per well, cultured for 24 h and incubated at 37°C with different samples for 24 h. FAM-aptPD-L1/Cy5-PD1 were subsequently added and the cells were detected using flow cytometry (CytoFLEX, Beckman Coulter).

Evaluation of IFN-γ/IL-10 expression levels in CD3+ T cells: 4T1 cells were inoculated into 12-well plate with 1.5×105 per well. The cells were cultured for 24 h, added with different samples and splenocytes of 30 times the amount of tumor cells, and then incubated at 37°C for 24 h. The splenocytes were collected and incubated with PC7-anti-CD45 antibody, APC-anti-CD3 antibody, FITC-anti-IFN-γ antibody and PE-anti-IL-10 antibody at room temperature for 30 min in the dark. Finally, the cells were detected by flow cytometry (CytoFLEX, Beckman Coulter).

Instrument	Flow cytometry (CytoFLEX, Beckman Coulter)
Software	FlowJo-V10 and CytExpert
Cell population abundance	The cells stained with different markers were filtered using 300 screen mesh cell strainer. 10000 cells were extracted from individual samples for flow cytometry analysis.
Gating strategy	Living cells are first gated on the basis of their scattering properties using forward (FSC) and side scatter (SSC). Adhered particles and impurities were removed via FSCA/FSCH approach. Immune cells were gated via APC/SSCA approach to differentiate T cells. Activation status of T cells was determined by CD4/CD8 or CD8/IFN-γ expressions.

☒ Tick this box to confirm that a figure exemplifying the gating strategy is provided in the Supplementary Information.
